# Supplementary material for: Surfactant Effect on the Physicochemical Characteristics of Solid Lipid Nanoparticles Based on Pillar[5]arenes
Source: Int J Mol Sci. 2022 Jan 11;23(2):779. doi: 10.3390/ijms23020779 (PMC8775580; doi:10.3390/ijms23020779)

Surfactant effect on the physicochemical characteristics of solid lipid nanoparticles based on pillar[5]arenes

Anastasia Nazarova*, Luidmila Yakimova*, Darya Filimonova, and Ivan Stoikov*

A.M.Butlerov Chemical Institute, Kazan Federal University, 420008 Kremlevskaya, 18, Kazan, Russian Federation; filimon.darya@gmail.com (D.F.).

*Correspondence: anas7tasia@gmail.com (A.N.); mila.yakimova@mail.ru (L.Y.); ivan.stoikov@mail.ru (I.S.)

Electronic Supplementary Information (33 pages)

Fig. S1. ^{1}H NMR spectrum of 4-(3-(3-carboxypropanamido)propoxy)-8,14,18,23,26,28,31,32,35-nonamethoxypillar[5]arene (4), $CDCl_3$, 298 K, 400 MHz.

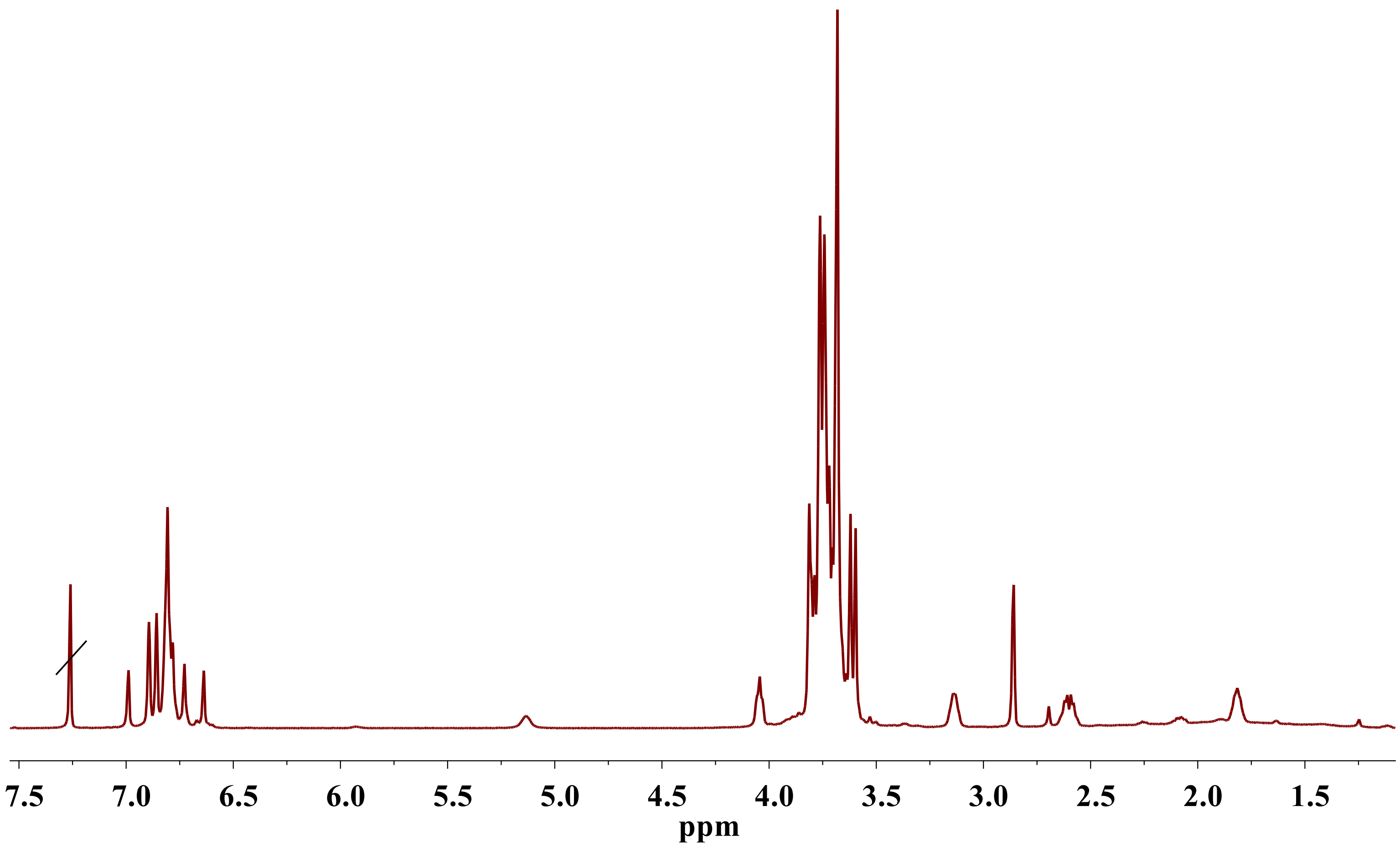

Fig. S2. ^{1}H NMR spectrum of 4-(3-(3-carboxypropanamido)propoxy)-8,14,18,23,26,28,31,32,35-nonamethoxypillar[5]arene (4), DMSO-d_6, 298 K, 400 MHz.

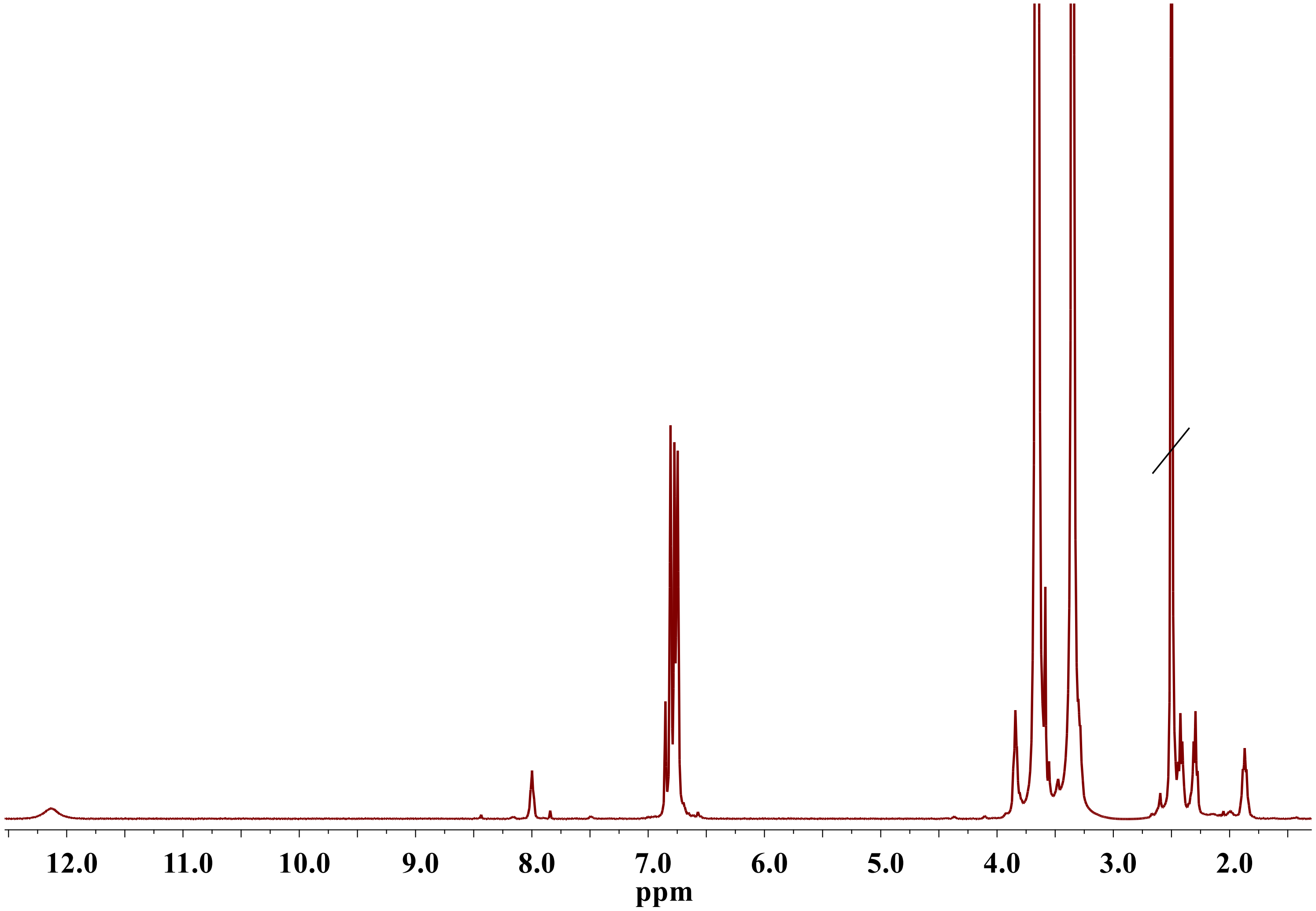

Fig. S3. ¹H NMR spectrum of 4-[(*N*-(3-carboxypropanamido)-{4′-aminobutyl}-amino)-carbamoylmethoxy]-8,14,18,23,26,28,31,32,35-nonamethoxy-pillar[5]arene (5), $CDCl_3$, 298 K, 400 MHz.

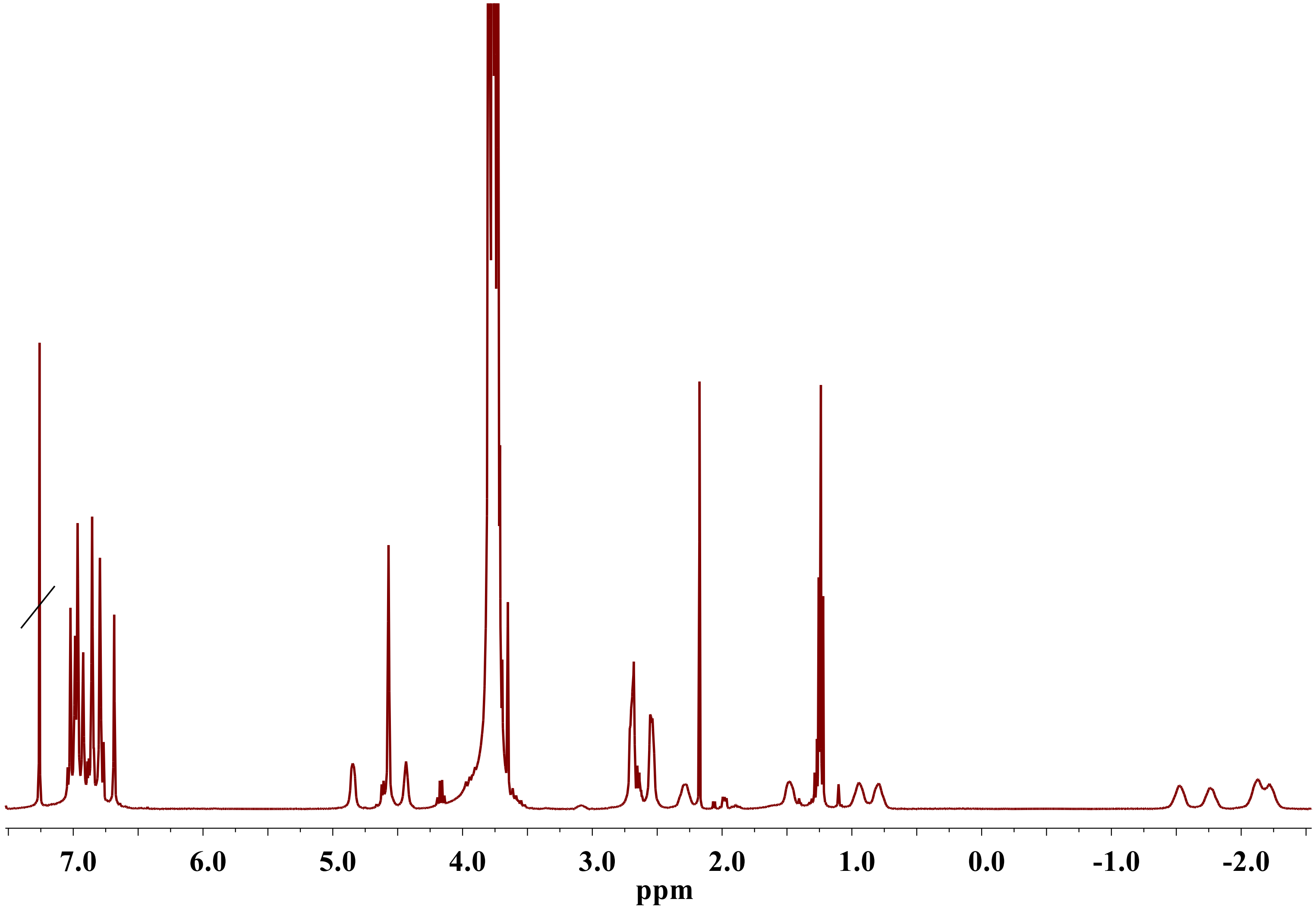

Fig. S4. ^{1}H NMR spectrum of 4-[(*N*-(3-carboxypropanamido)-{4′-aminobutyl}-amino)-carbamoylmethoxy]-8,14,18,23,26,28,31,32,35-nonamethoxy-pillar[5]arene (5), DMSO-d_6, 298 K, 400 MHz.

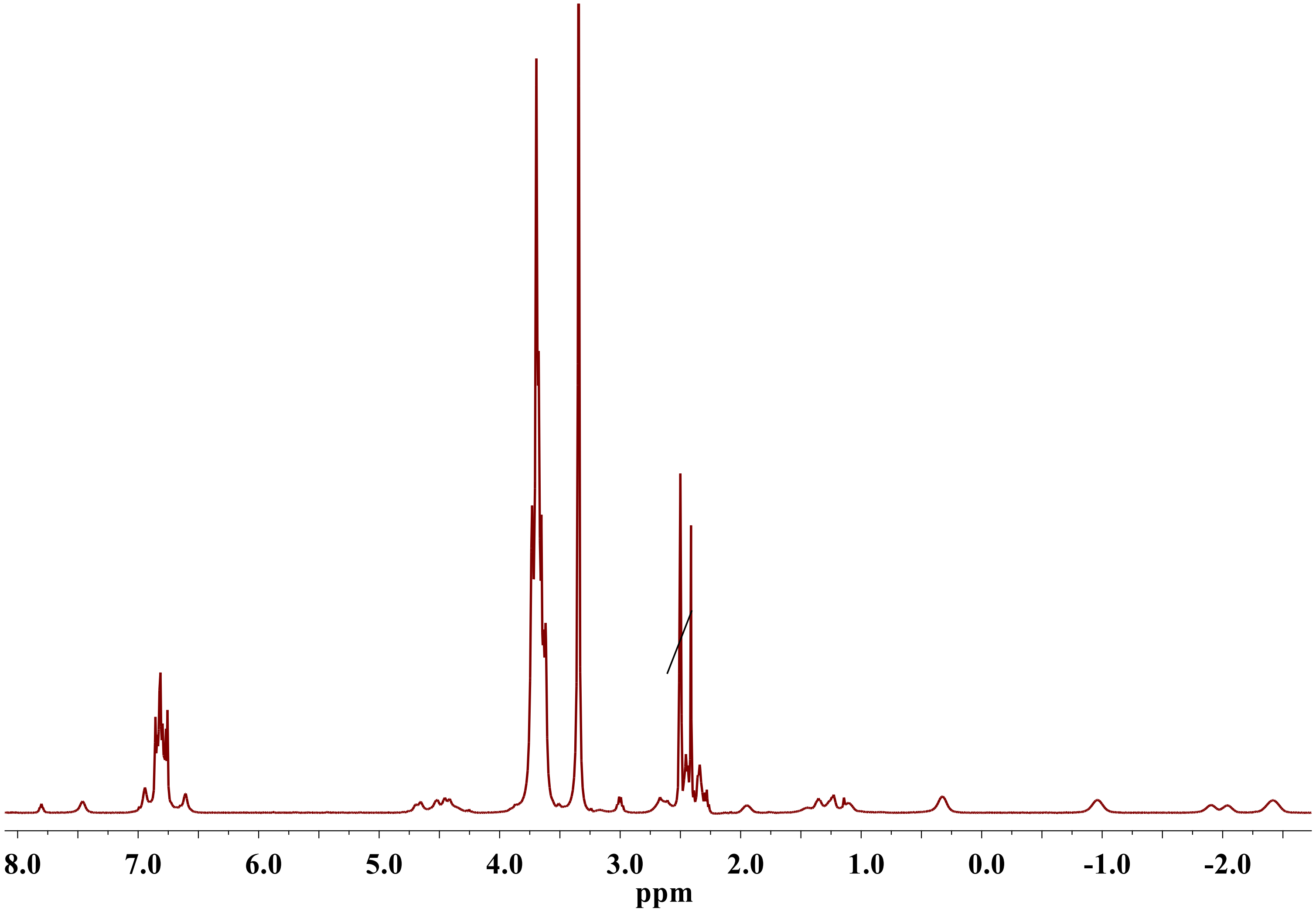

Fig. S5. ^{1}H NMR spectrum of 4-[(*N*-(3-carboxypropanamido)-{6′-aminohexyl}-amino)-carbamoylmethoxy]-8,14,18,23,26,28,31,32,35-nonamethoxypillar[5]arene (6), $CDCl_3$, 298 K, 400 MHz.

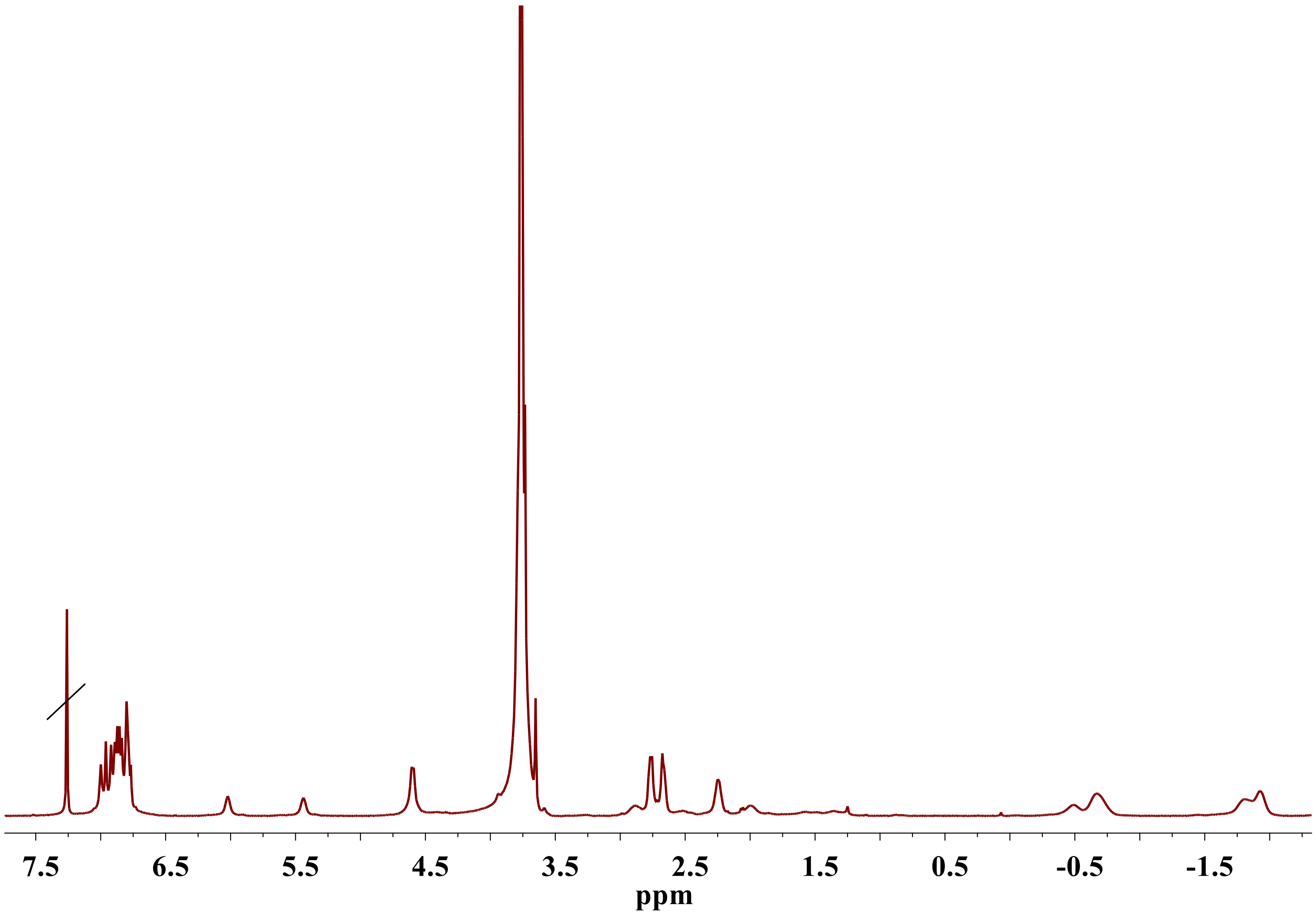

Fig. S6. [1]**H NMR spectrum of 4-[(*N*-(3-carboxypropanamido)-{6′-aminohexyl}-amino)-carbamoylmethoxy]-8,14,18,23,26,28,31,32,35-nonamethoxypillar[5]arene (6), DMSO-d_6, 298 K, 400 MHz.**

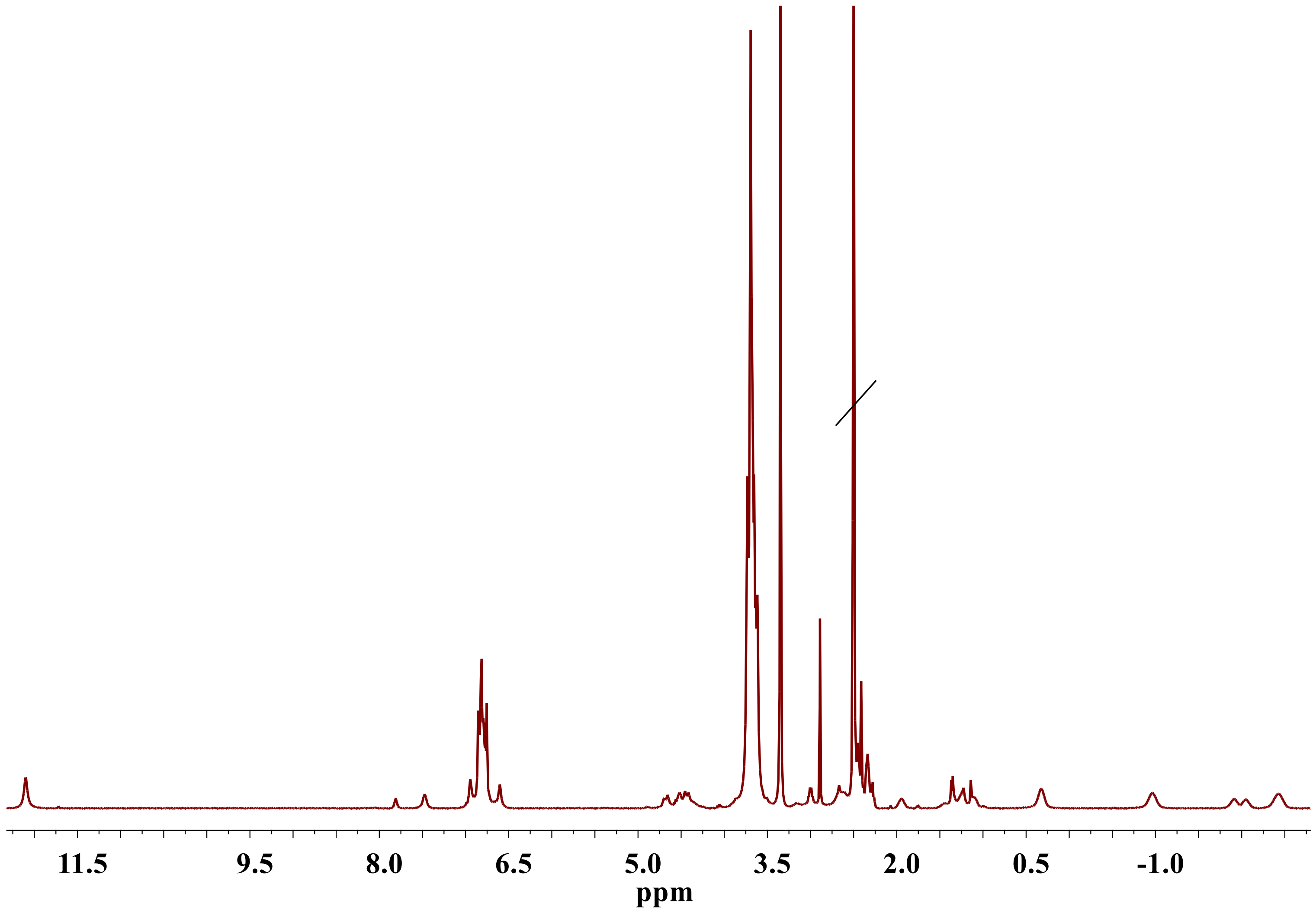

Fig. S7. ^{13}**C NMR spectrum of 4-(3-(3-carboxypropanamido)propoxy)-8,14,18,23,26,28,31,32,35-nonamethoxypillar[5]arene (4), DMSO-*d*$_6$, 298 K, 100 MHz.**

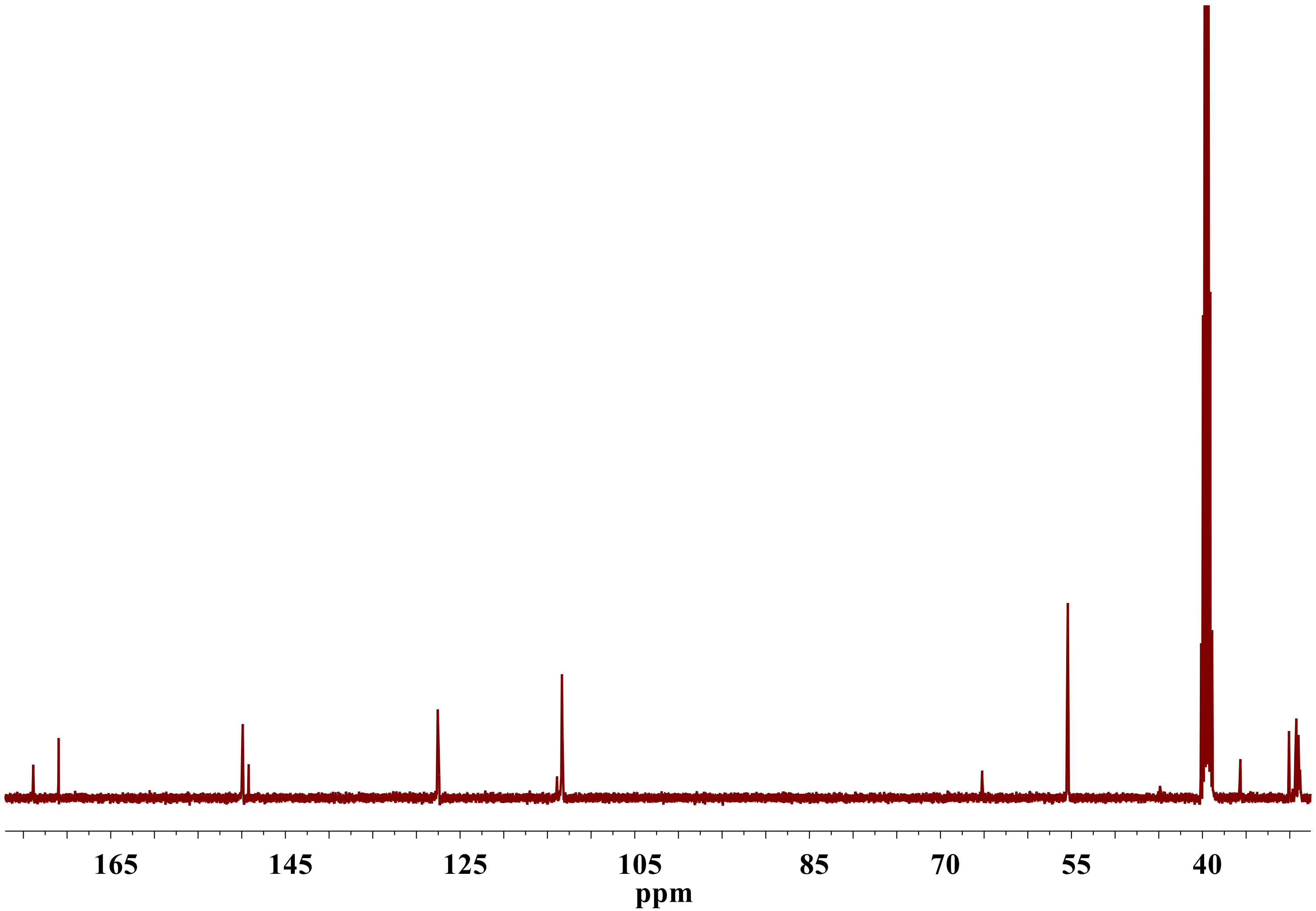

Fig. S8. ^{13}**C NMR spectrum of 4-[(*N*-(3-carboxypropanamido)-{4′-aminobutyl}-amino)-carbamoylmethoxy]-8,14,18,23,26,28,31,32,35-nonamethoxy-pillar[5]arene (5), DMSO-*d*$_6$, 298 K, 100 MHz.**

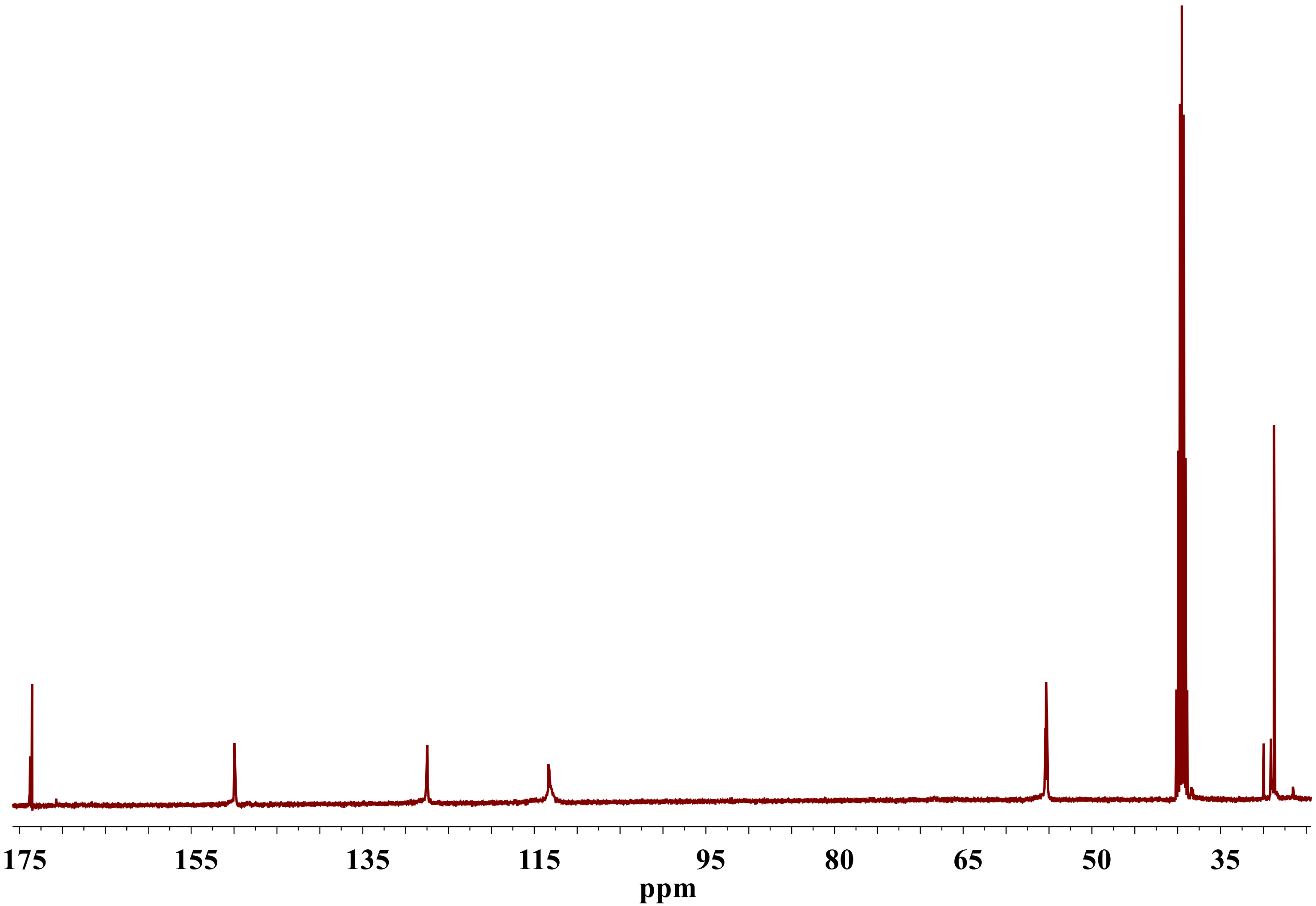

Fig. S9. [13]**C NMR spectrum of 4-[(*N*-(3-carboxypropanamido)-{6′-aminohexyl}-amino)-carbamoylmethoxy]-8,14,18,23,26,28,31,32,35-nonamethoxypillar[5]arene (6), DMSO-*d*₆, 298 K, 100 MHz.**

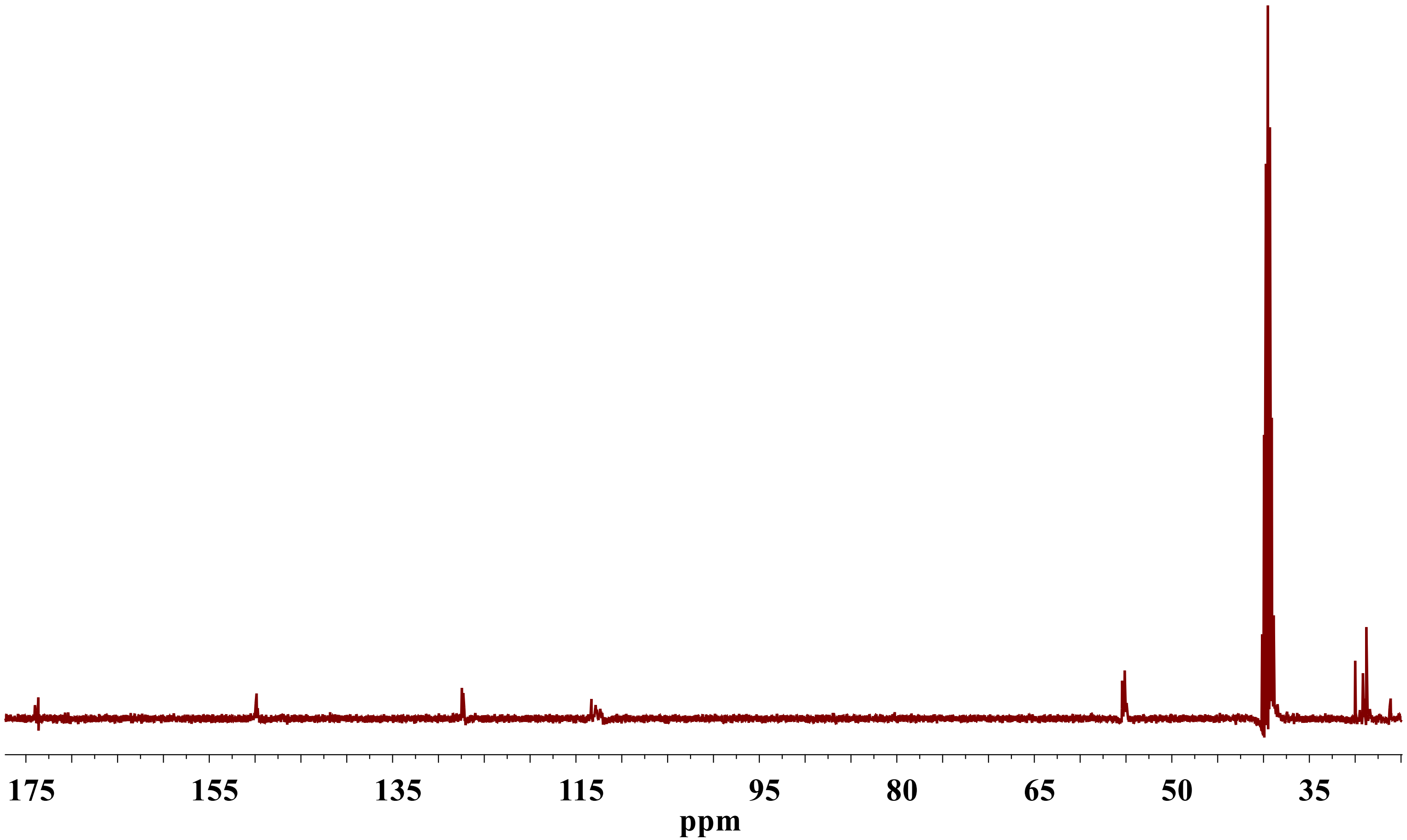

Fig. S10. Mass spectrum (MALDI) of 4-(3-(3-carboxypropanamido)propoxy)-8,14,18,23,26,28,31,32,35-nonamethoxypillar[5]arene (4).

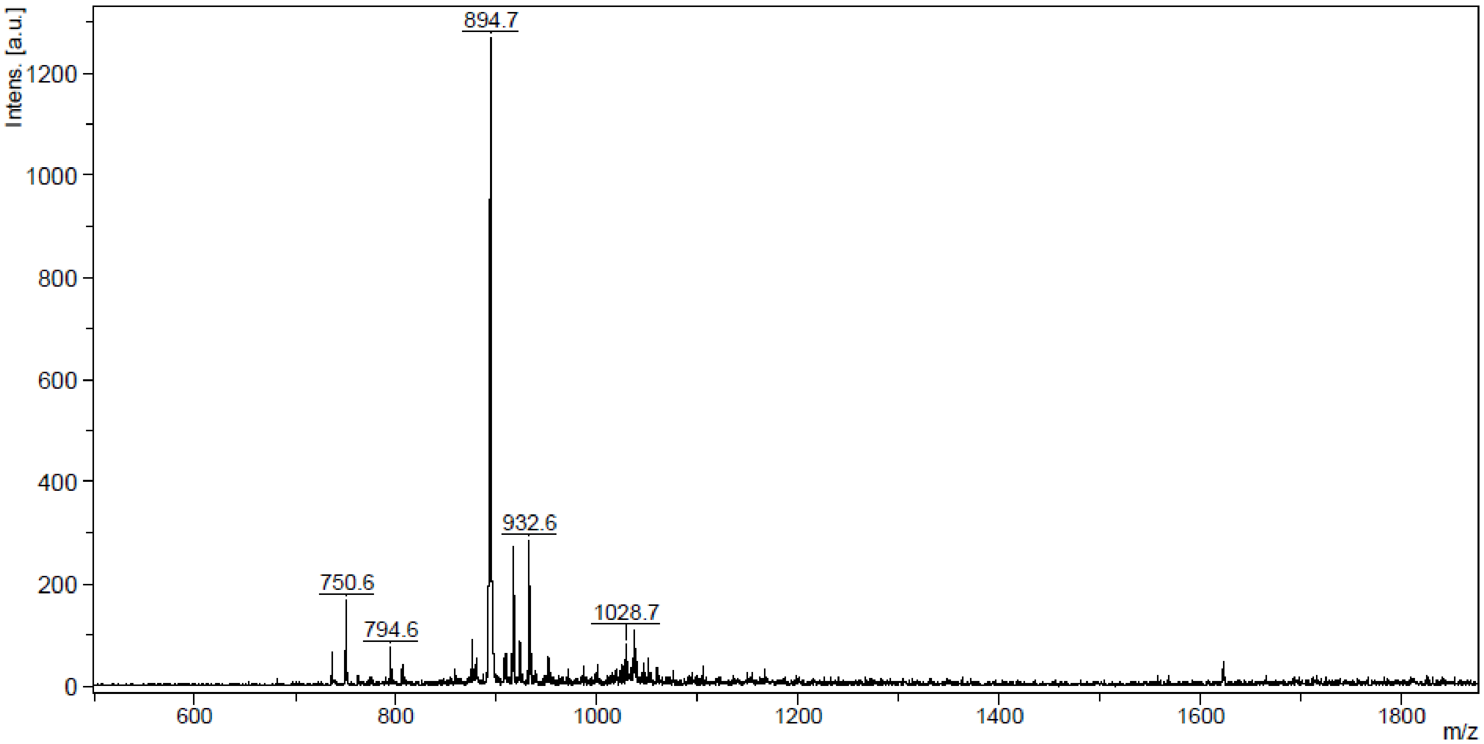

**Instrument**	
Instrument type	ultraflexTOF/TOF
Name of computer	MALDI
flexControl version	flexControl 3.0.173.0
flexAnalysis version	3.0.96.0
**Spectrometer**	
Ion Polarity	POS
PIE delay	0 ns
Ion source voltage 1	25 kV
Ion source voltage 2	23.65 kV
Lens voltage	6 kV
Linear detector voltage	1.405 kV
Reflector voltage 1	0 kV
Reflector voltage 2	0 kV
Reflector detector voltage	1.569 kV
**Laser**	
Ion Source Type	MALDI
Laser Type	Nd:YAG
Wavelength	355 nm
Number of shots	50
Laser repetition rate	100 Hz
**Target**	
Target Plate	MTP AnchorChip
Position	P24

Fig. S11. Mass spectrum (MALDI) of 4-[(*N*-(3-carboxypropanamido)-{4′-aminobutyl}-amino)-carbamoylmethoxy]-8,14,18,23,26,28,31,32,35- nonamethoxy-pillar[5]arene (5).

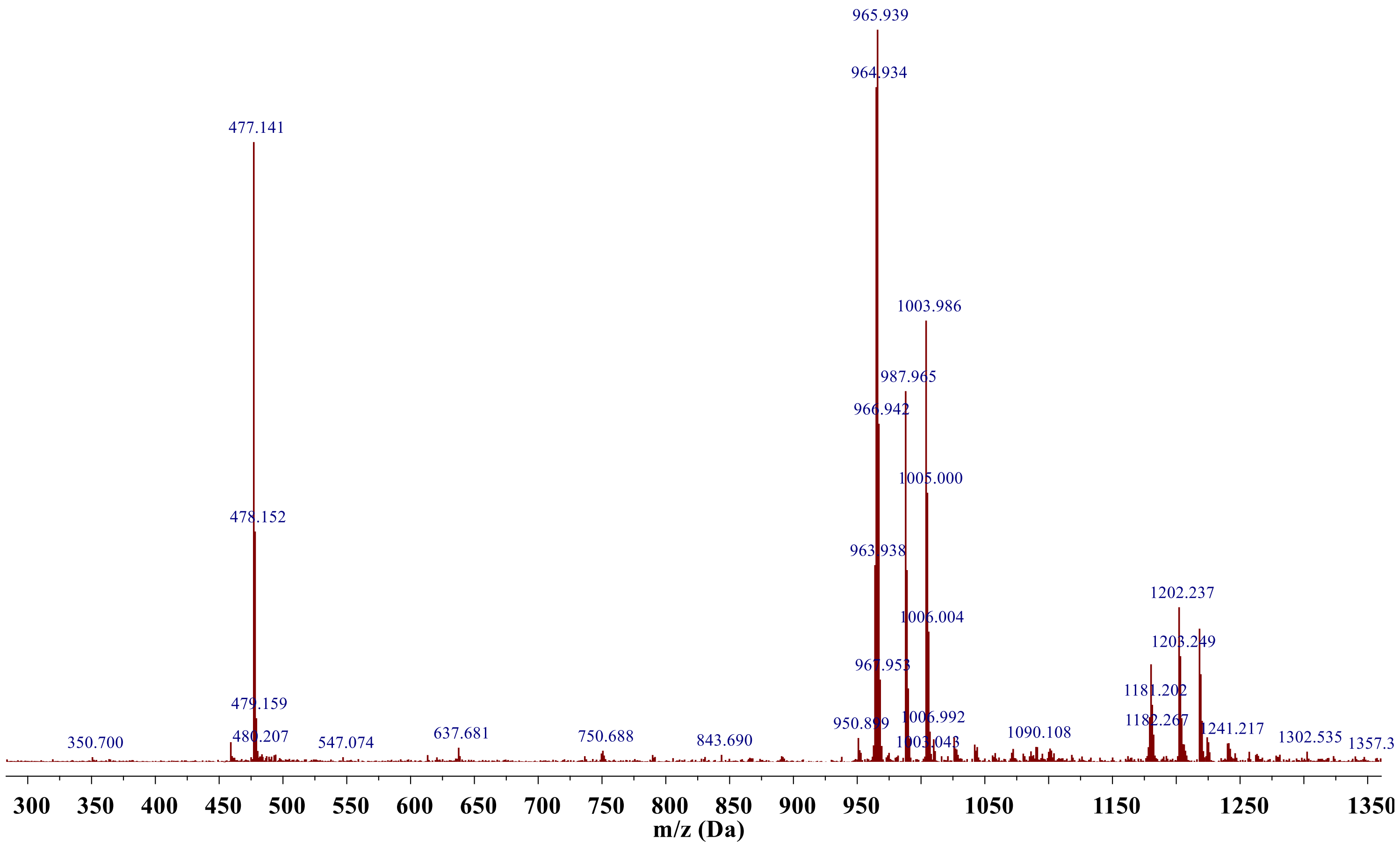

Fig. S12. Mass spectrum (MALDI) of 4-[(*N*-(3-carboxypropanamido)-{6′-aminohexyl}-amino)-carbamoylmethoxy]-8,14,18,23,26,28,31,32,35-nonamethoxypillar[5]arene (6).

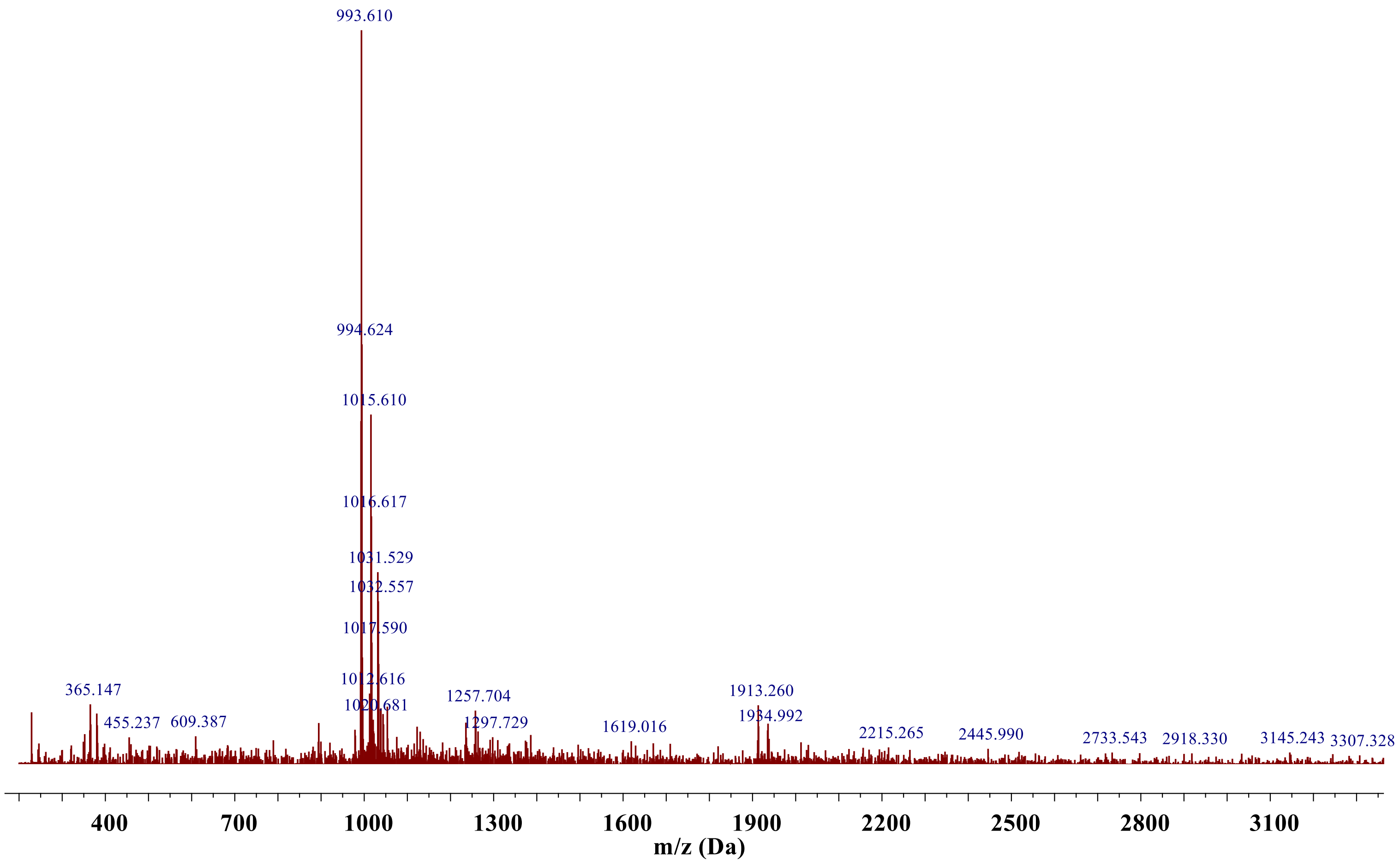

Fig. S13. IR spectrum of 4-(3-(3-carboxypropanamido)propoxy)-8,14,18,23,26,28,31,32,35-nonamethoxypillar[5]arene (4).

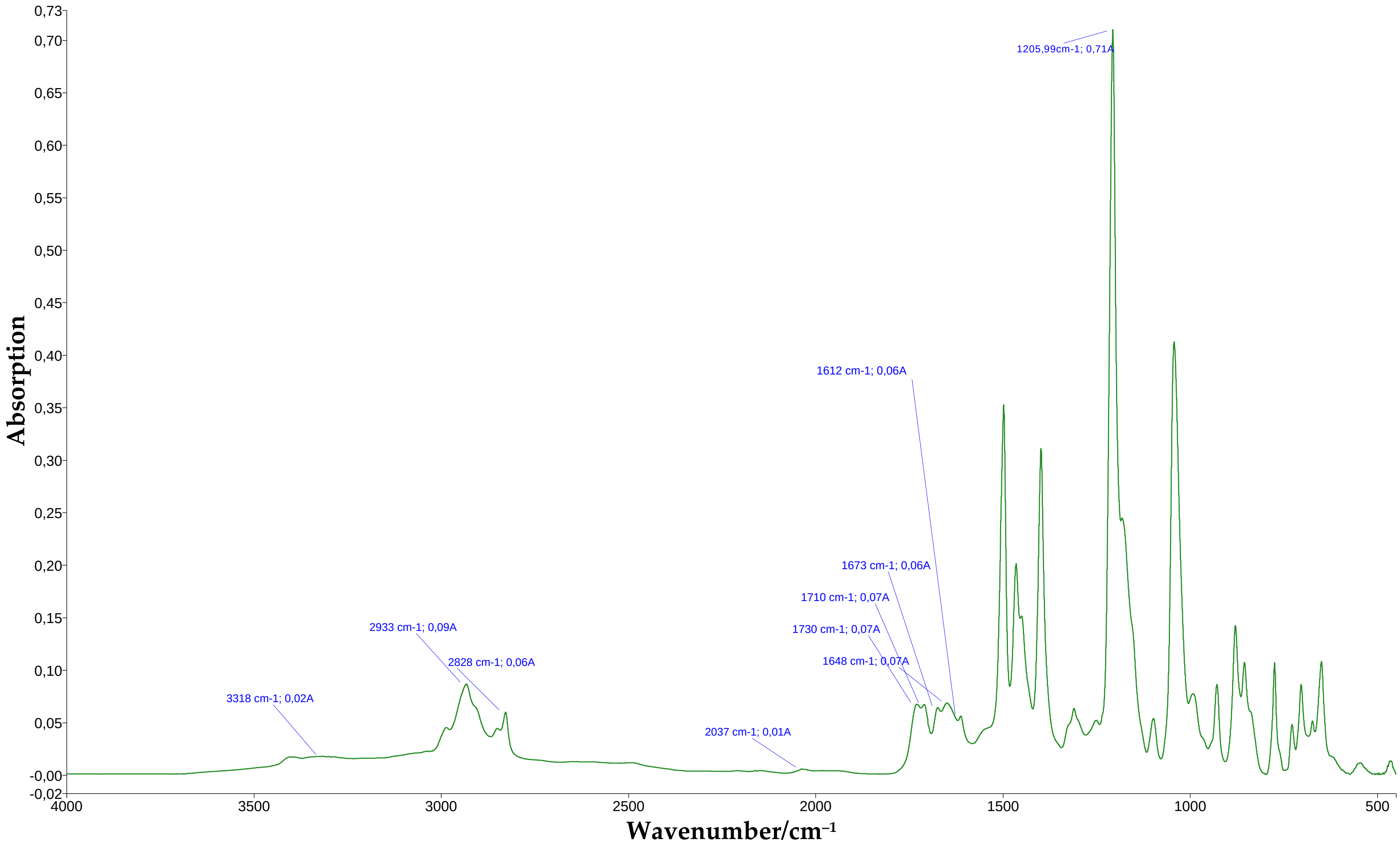

Fig. S14. IR spectrum of 4-[(*N*-(3-carboxypropanamido)-{4′-aminobutyl}-amino)-carbamoylmethoxy]-8,14,18,23,26,28,31,32,35-nonamethoxy-pillar[5]arene (5).

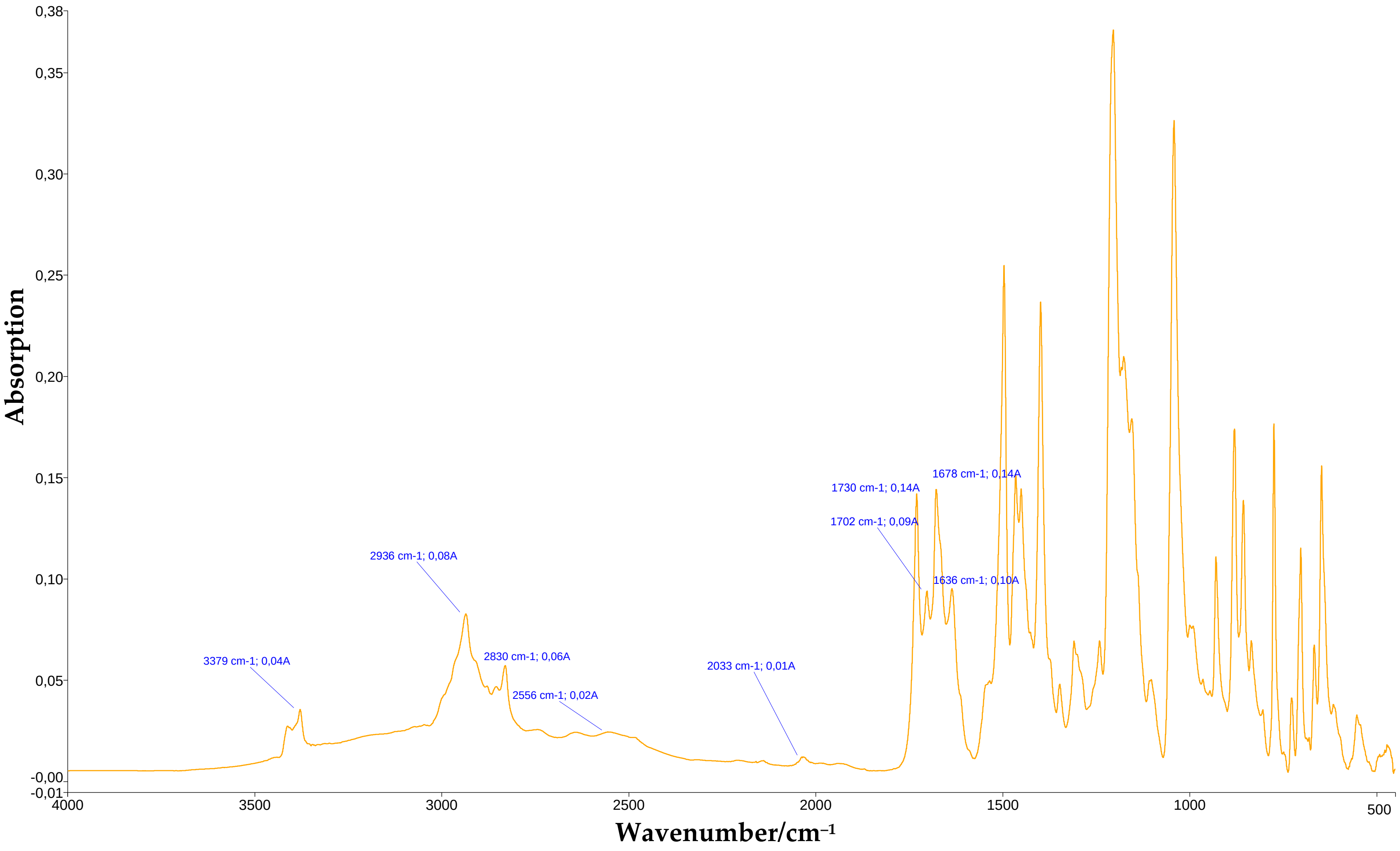

Fig. S15. IR spectrum of 4-[(*N*-(3-carboxypropanamido)-{6′-aminohexyl}-amino)-carbamoylmethoxy]-8,14,18,23,26,28,31,32,35-nonamethoxypillar[5]arene (6).

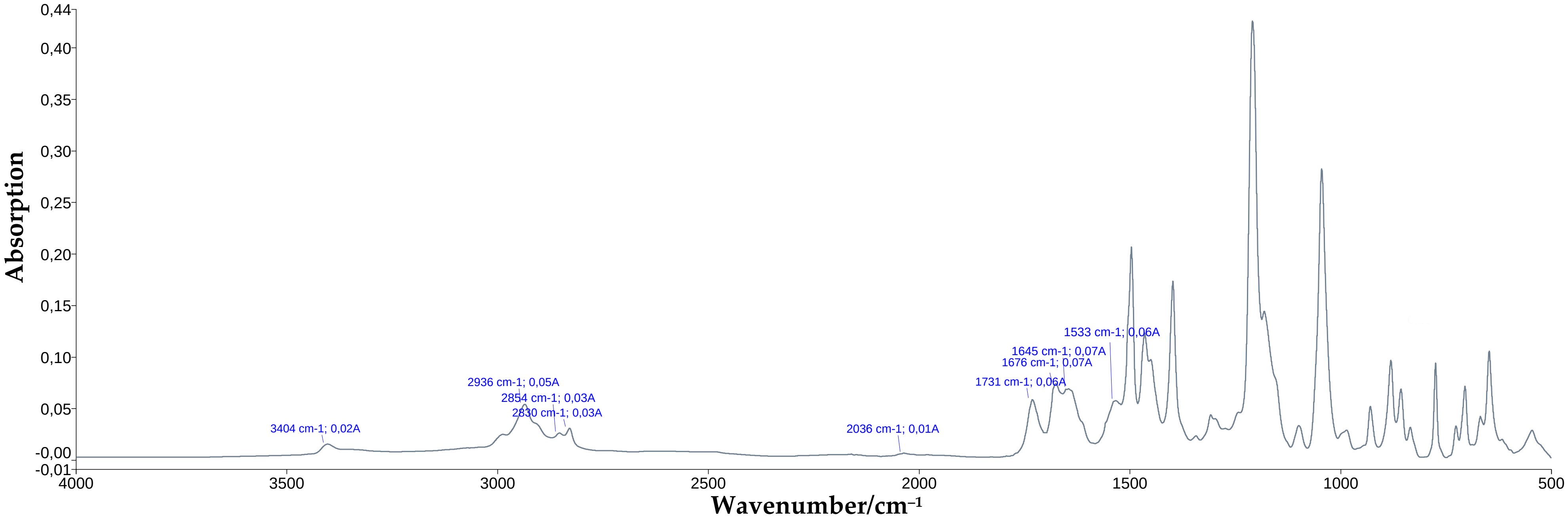

Fig. S16. Size distribution of particles by intensity for SLN-4 (3 × 10^{-4} M) in water (the each colored line on the graph means one repeated measurement).

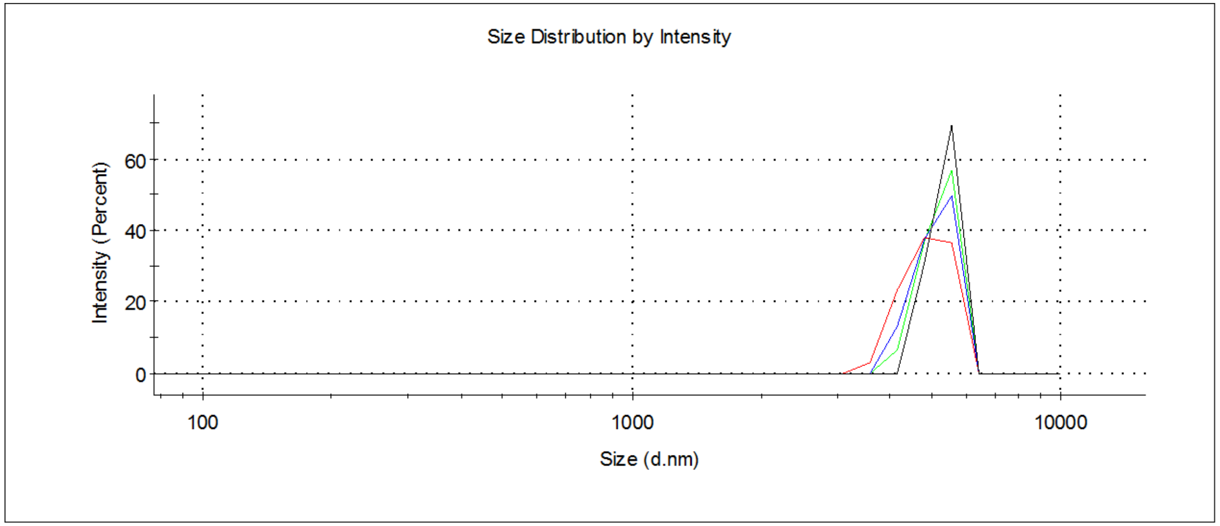

Fig. S17. Size distribution of particles by intensity for SLN-4 (1 × 10^{-4} M) in water (the each colored line on the graph means one repeated measurement).

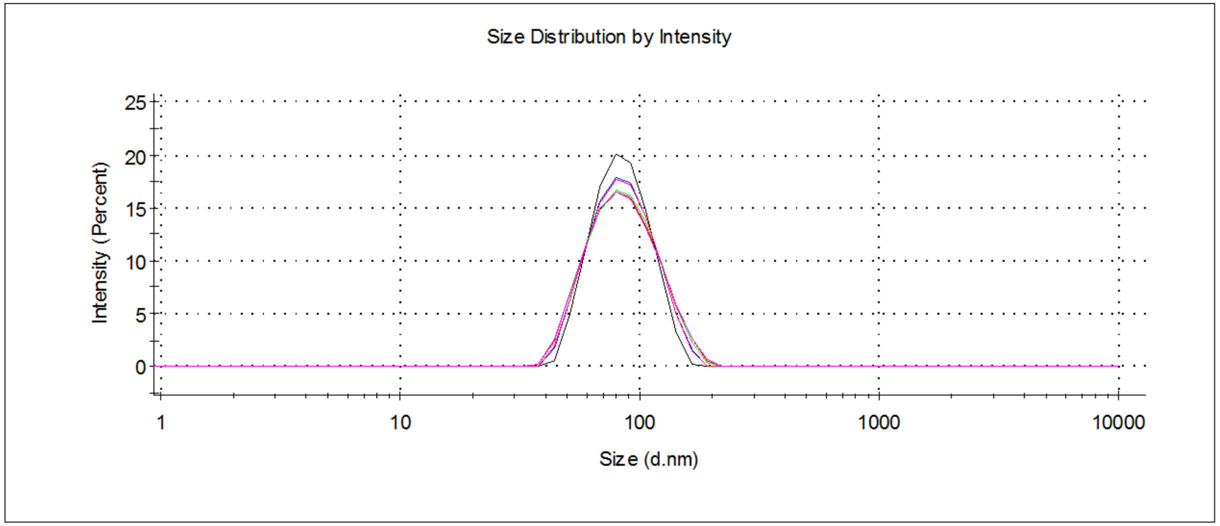

Fig. S18. Zeta potential distribution of the particles SLN-4 (1 × 10^{-4} M) in water (the each colored line on the graph means one repeated measurement).

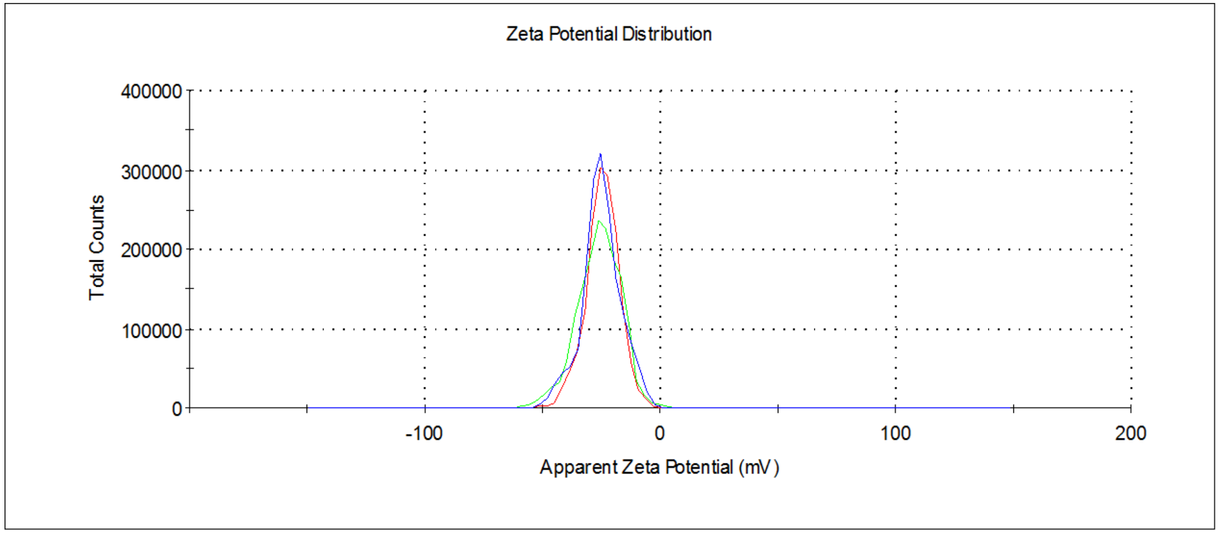

Fig. S19. Size distribution of particles by intensity for SLN-4 (5 × 10^{-5} M) in water (the each colored line on the graph means one repeated measurement).

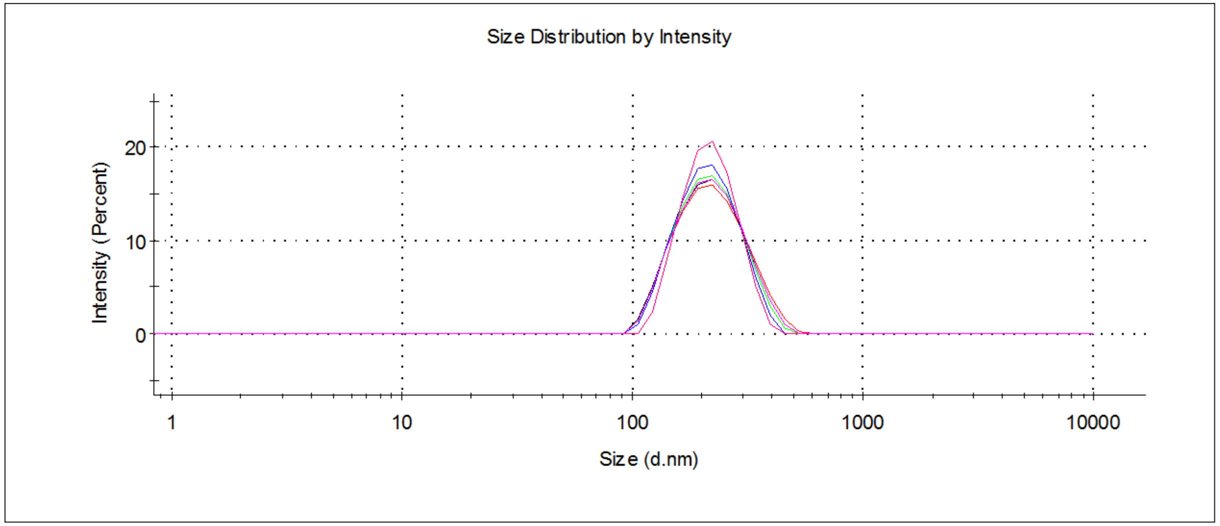

Fig. S20. Zeta potential distribution of the particles SLN-4 (5 × 10^{-5} M) in water (the each colored line on the graph means one repeated measurement).

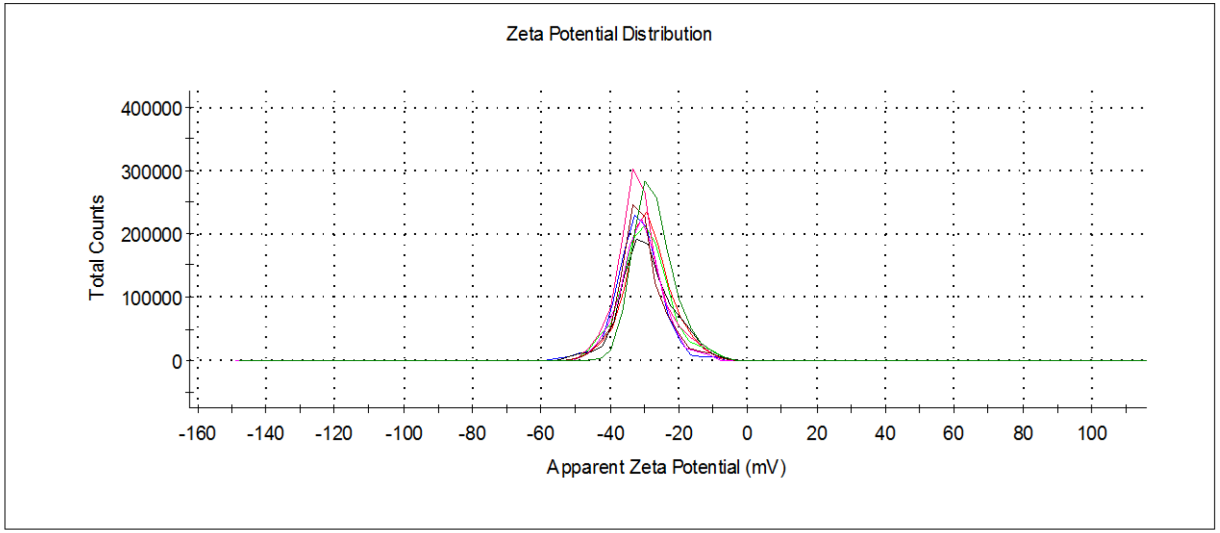

Fig. S21. Size distribution of particles by intensity for SLN-5 (5 × 10^{-5} M) in water (the each colored line on the graph means one repeated measurement).

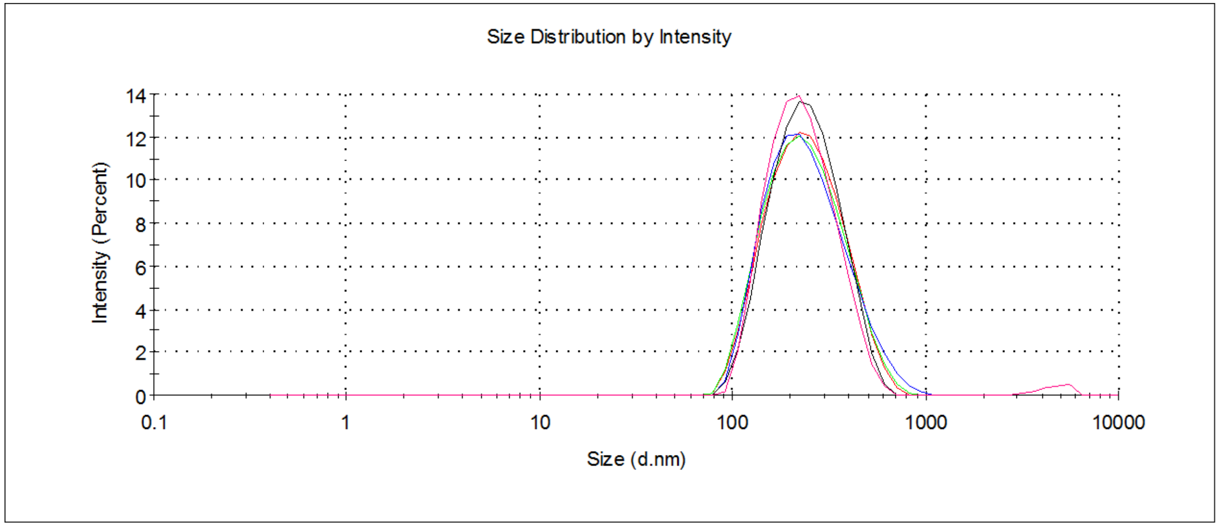

Fig. S22. Zeta potential distribution of the particles for SLN-5 (5 × 10^{-5} M) in water (the each colored line on the graph means one repeated measurement).

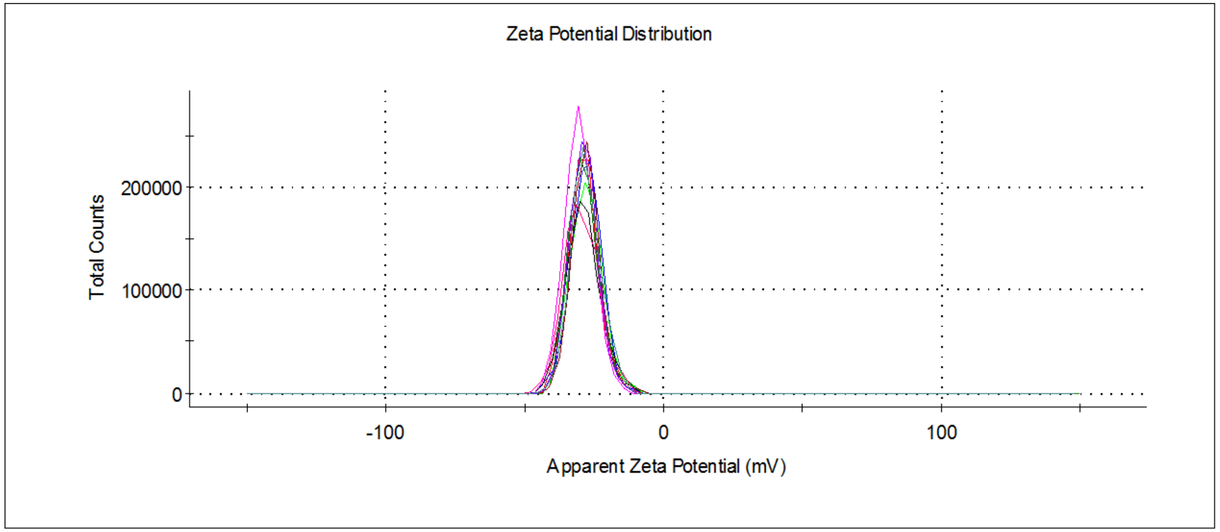

Fig. S23. Size distribution of particles by intensity for SLN-5 (1×10^{-4} M) in water (the each colored line on the graph means one repeated measurement).

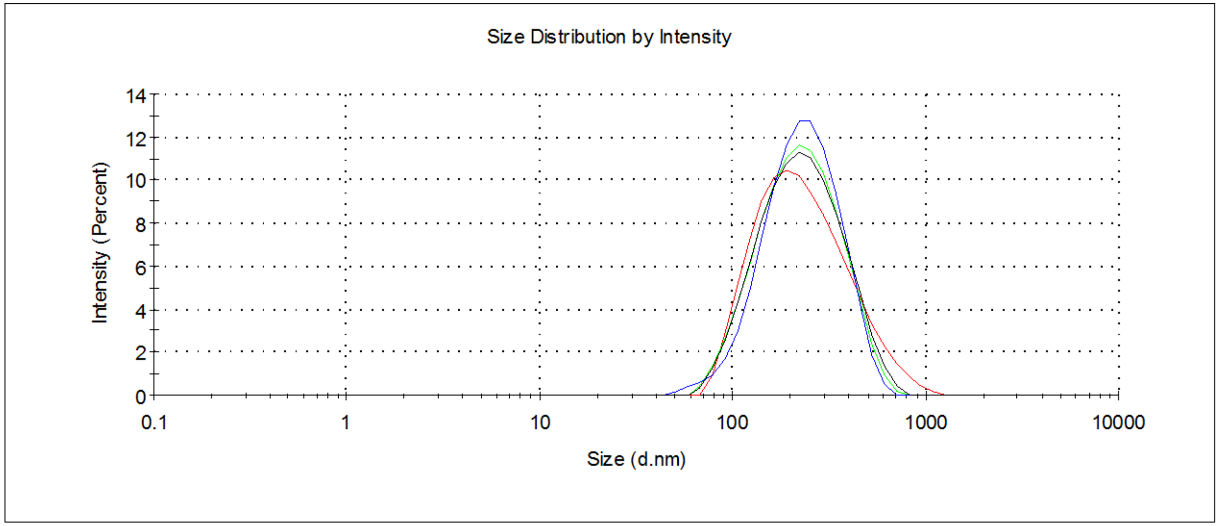

Fig. S24. Zeta potential distribution of the particles for SLN-5 (1×10^{-4} M) in water (the each colored line on the graph means one repeated measurement).

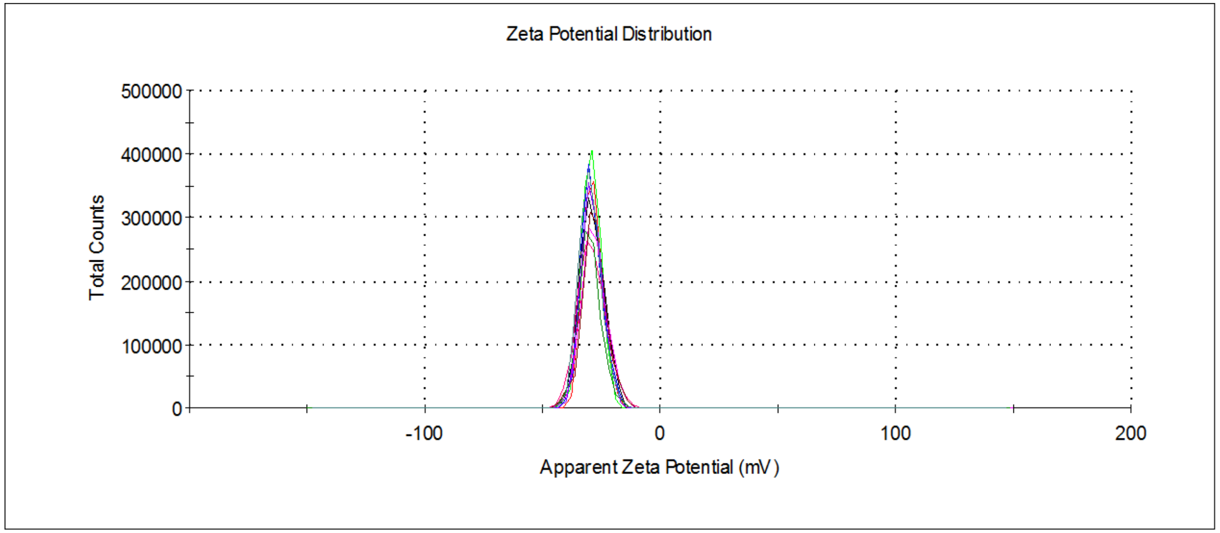

Fig. S25. Size distribution of particles by intensity for SLN-5 (3×10^{-4} M) in water (the each colored line on the graph means one repeated measurement).

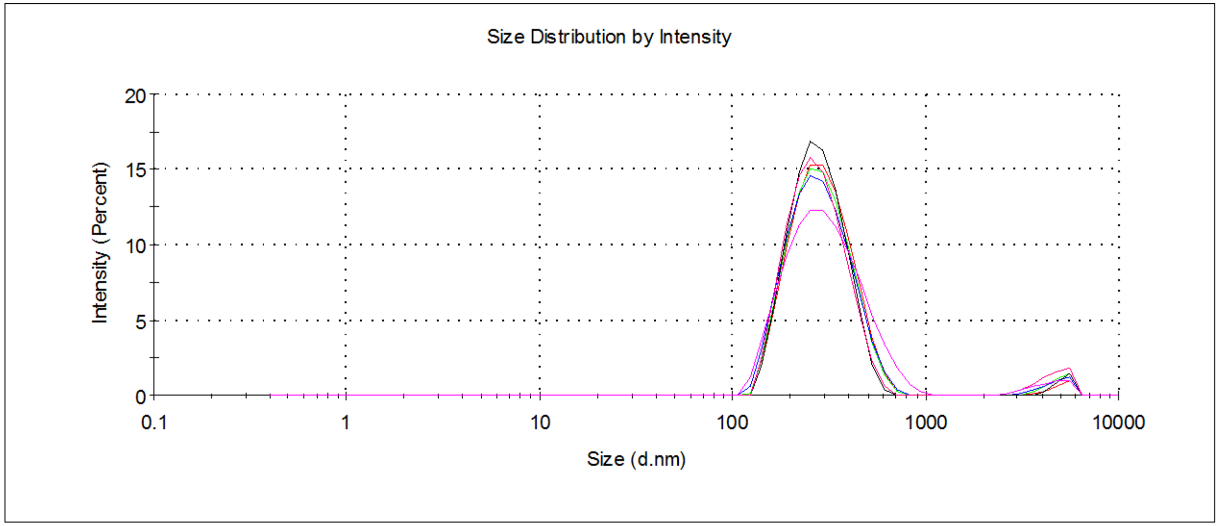

Fig. S26. Zeta potential distribution of the particles for SLN-5 (3×10^{-4} M) in water (the each colored line on the graph means one repeated measurement).

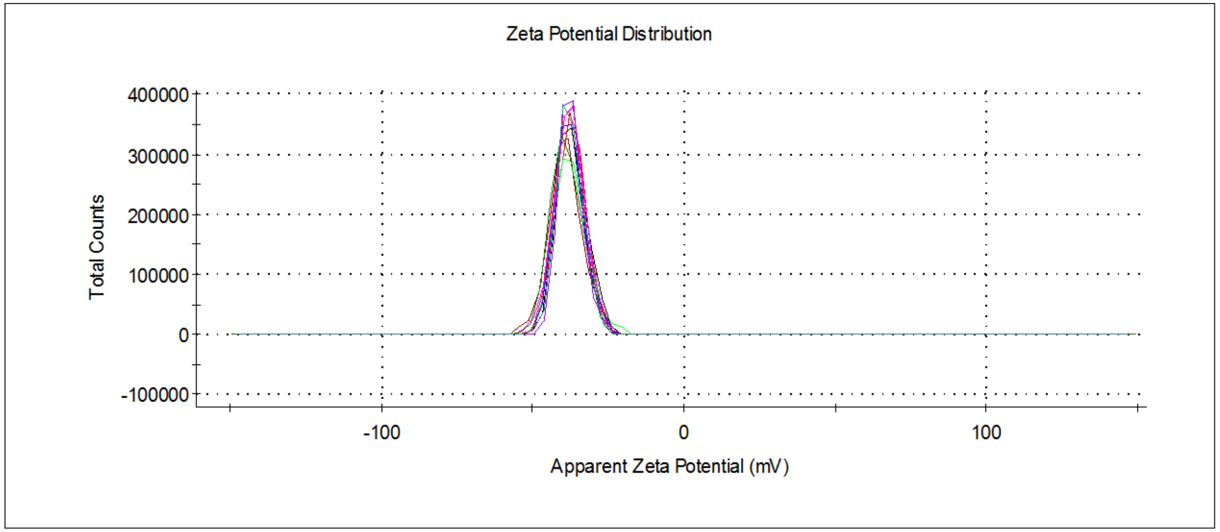

Fig. S27. Size distribution of particles by intensity for SLN-6 (5 × 10^{-5} M) in water (the each colored line on the graph means one repeated measurement).

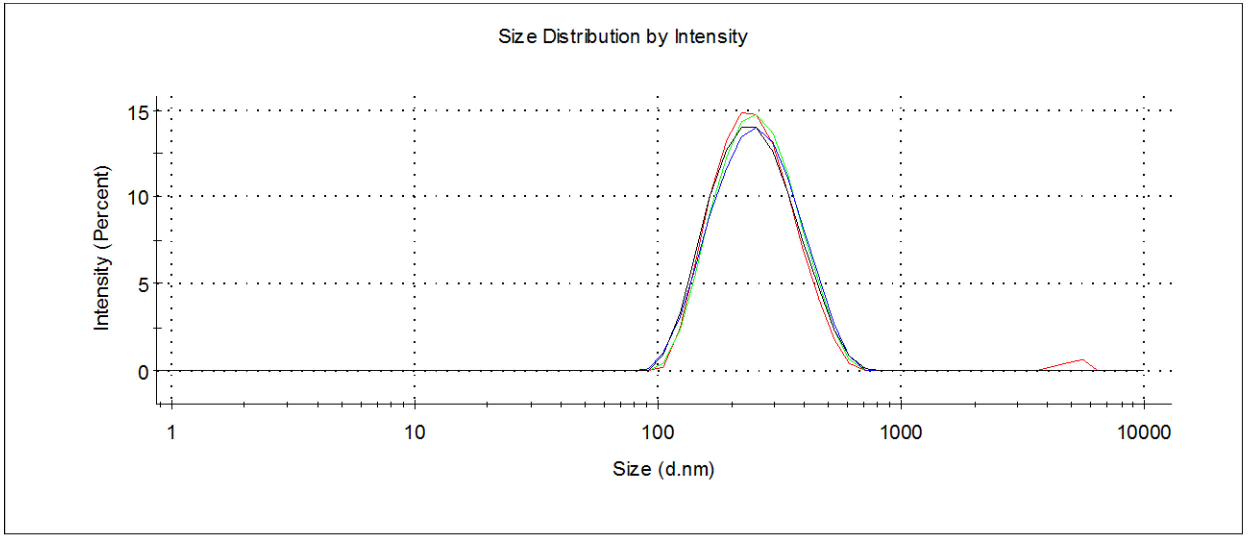

Fig. S28. Zeta potential distribution of the particles for SLN-6 (5 × 10^{-5} M) in water (the each colored line on the graph means one repeated measurement).

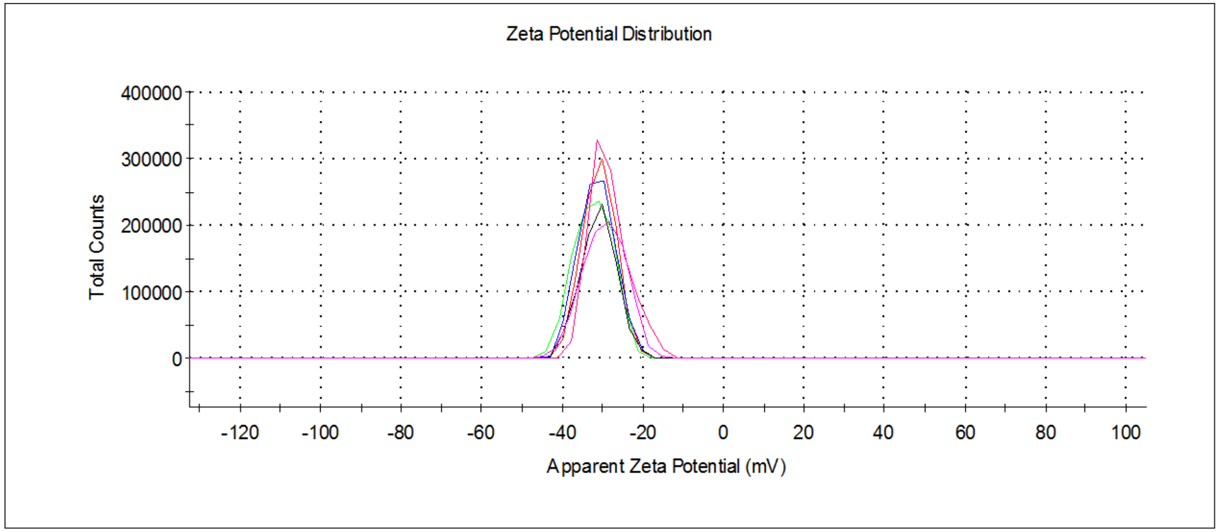

Fig. S29. Size distribution of particles by intensity for SLN-6 (1 × 10^{-4} M) in water (the each colored line on the graph means one repeated measurement).

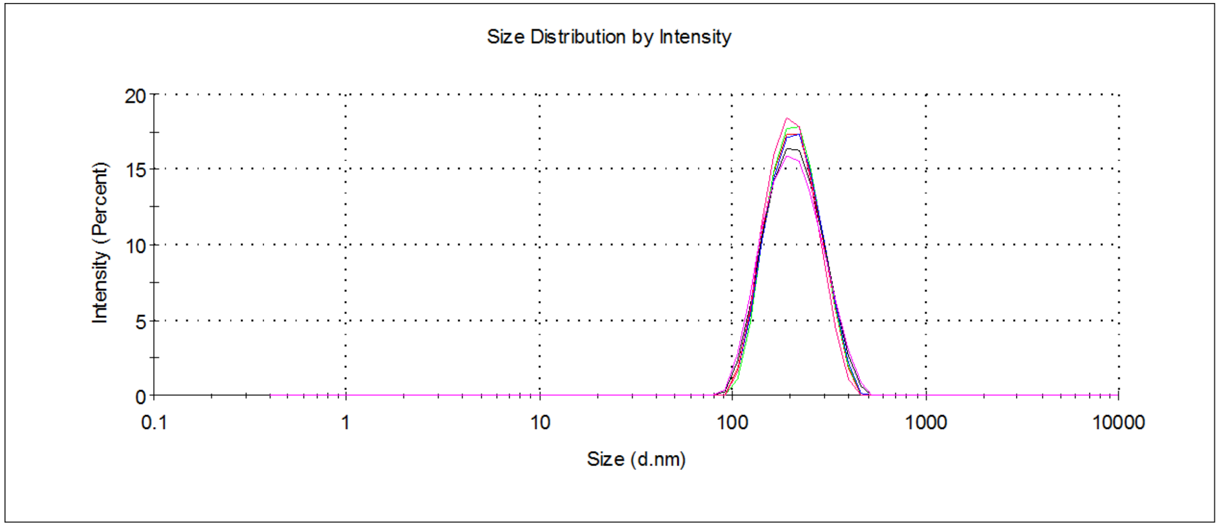

Fig. S30. Zeta potential distribution of the particles for SLN-6 (1 × 10^{-4} M) in water (the each colored line on the graph means one repeated measurement).

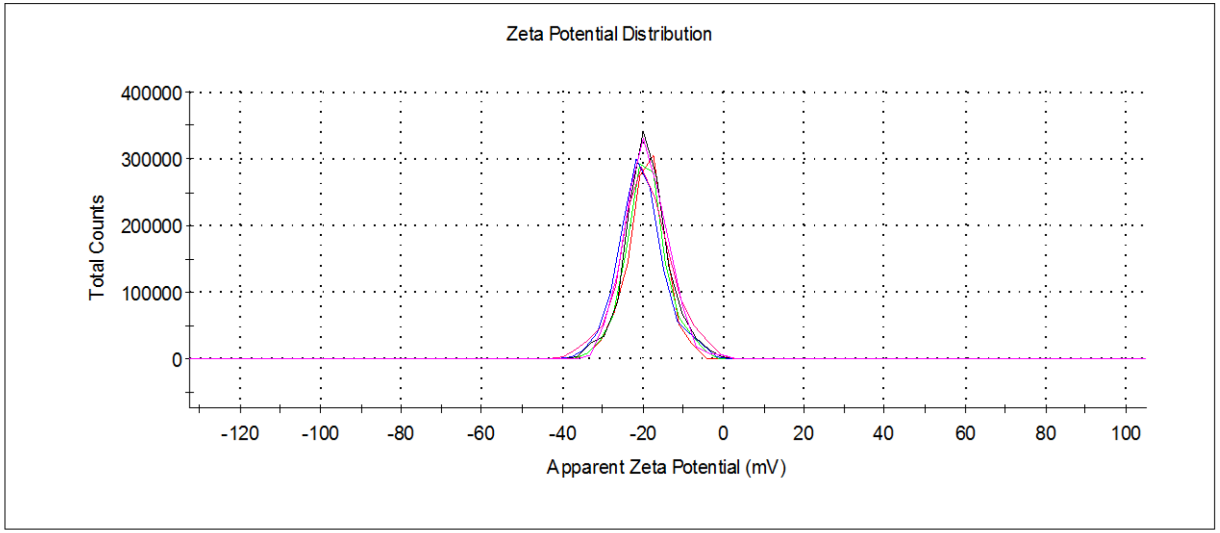

Fig. S31. Size distribution of particles by intensity for SLN-6 (3×10^{-4} M) in water (the each colored line on the graph means one repeated measurement).

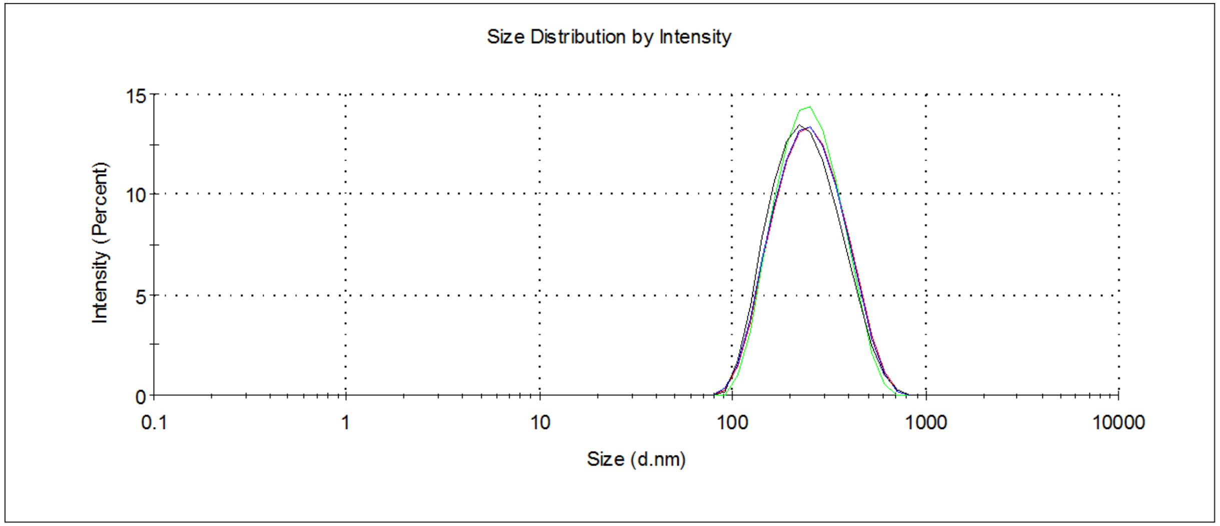

Fig. S32. Zeta potential distribution of the particles for SLN-6 (3×10^{-4} M) in water (the each colored line on the graph means one repeated measurement).

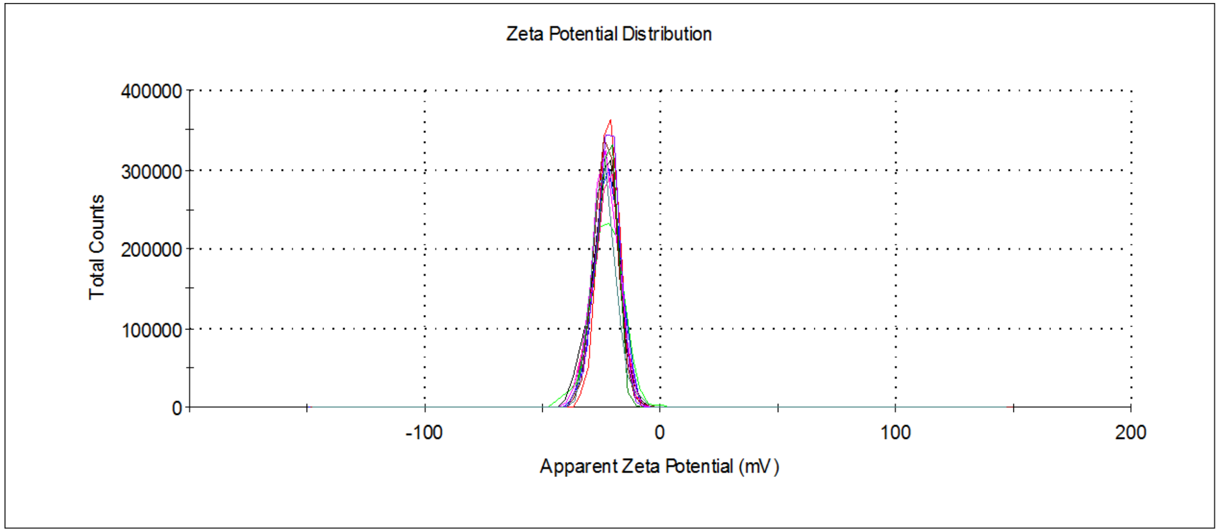

Fig. S33. Size distribution of the particles by intensity for SLN-4-DTAC (1 × 10^{-4} M) at 1:1 ratio in water (the each colored line on the graph means one repeated measurement).

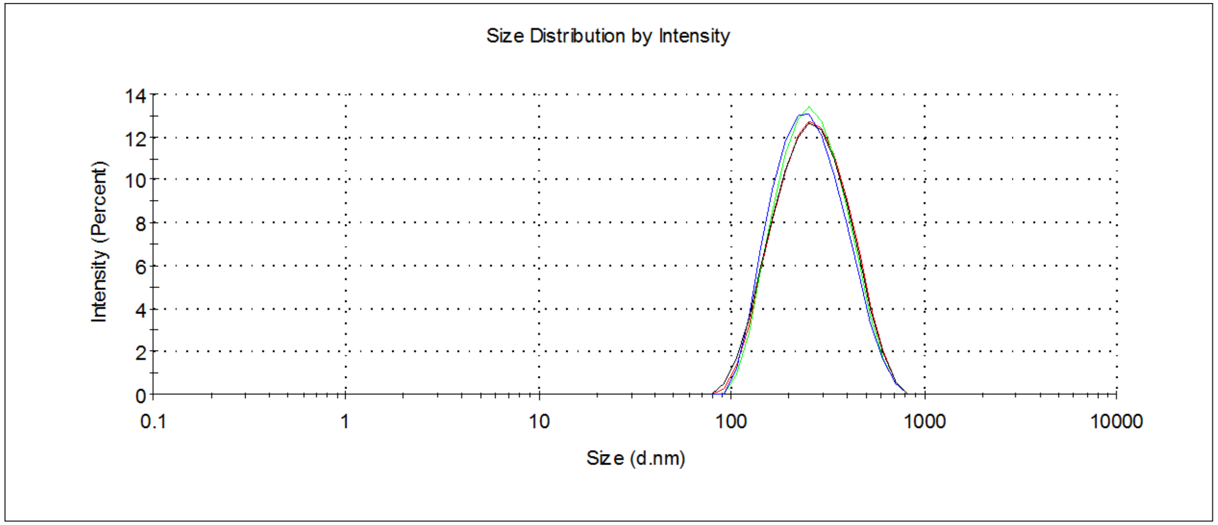

Fig. S34. Zeta potential distribution of the particles for SLN-4-DTAC (1 × 10^{-4} M) at 1:1 ratio in water (the each colored line on the graph means one repeated measurement).

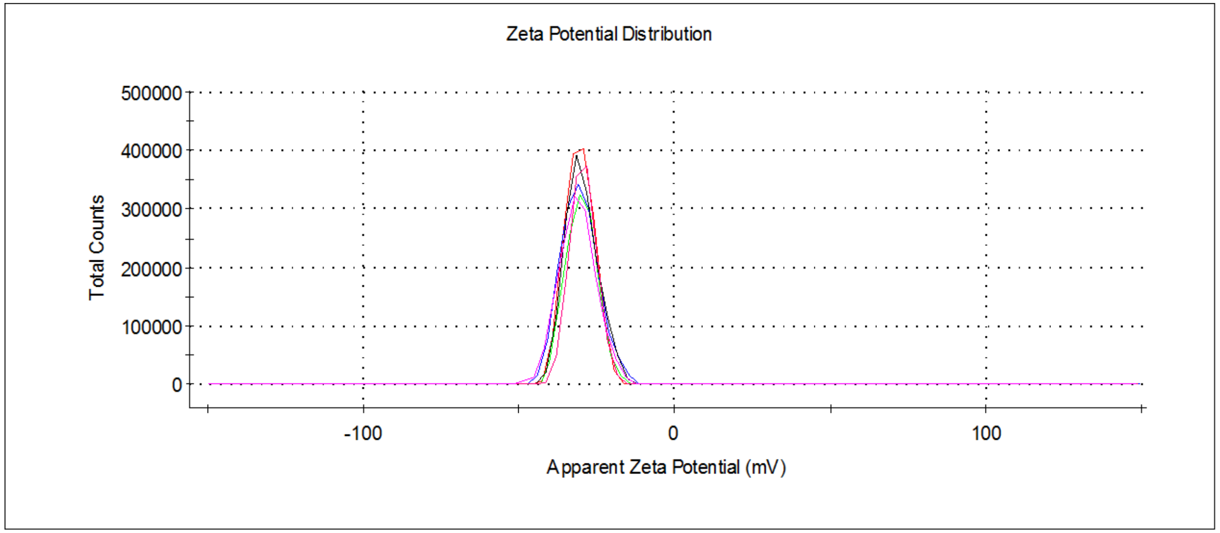

Fig. S35. Size distribution of the particles by intensity for SLN-4-DTAC (3 × 10^{-4} M) at 1:1 ratio in water (the each colored line on the graph means one repeated measurement).

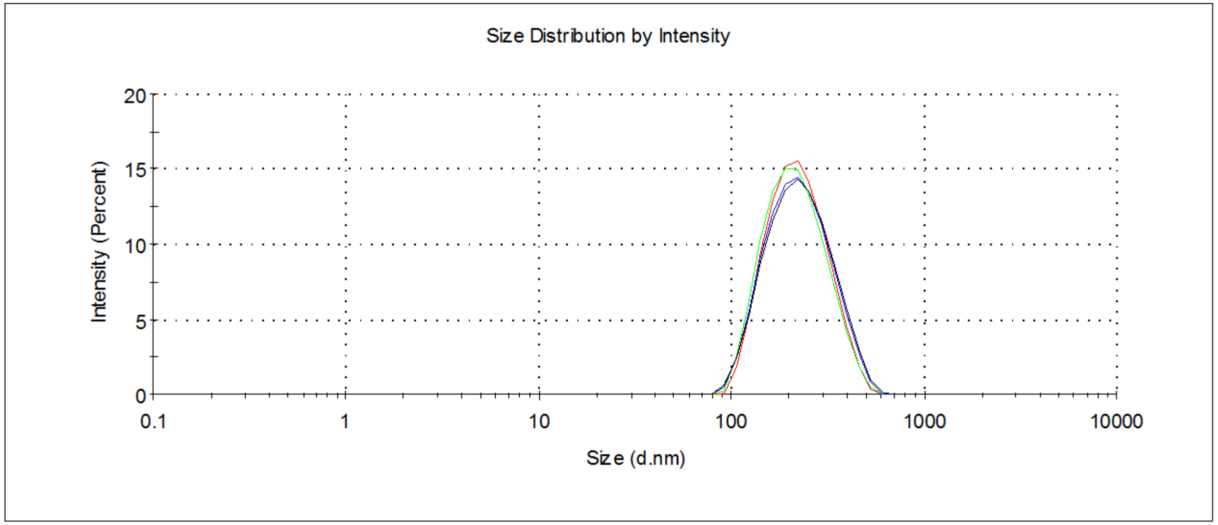

Fig. S36. Zeta potential distribution of the particles for SLN-4-DTAC (3 × 10^{-4} M) at 1:1 ratio in water (the each colored line on the graph means one repeated measurement).

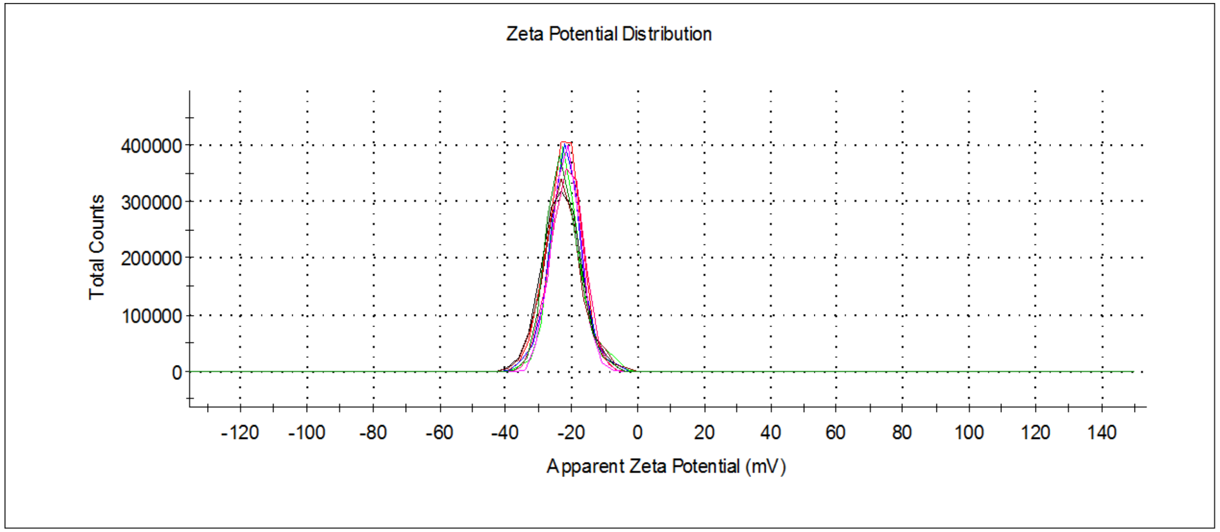

Fig. S37. Size distribution of the particles by intensity for SLN-5-DTAC (1×10^{-4} M) at 1:1 ratio in water (the each colored line on the graph means one repeated measurement).

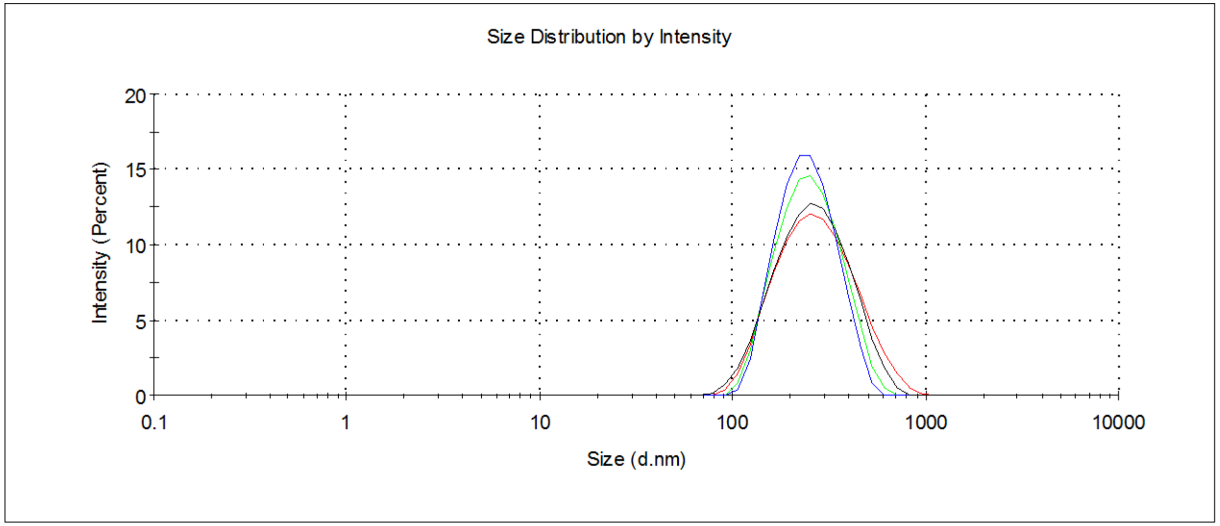

Fig. S38. Zeta potential distribution of the particles for SLN-5-DTAC (1×10^{-4} M) at 1:1 ratio in water (the each colored line on the graph means one repeated measurement).

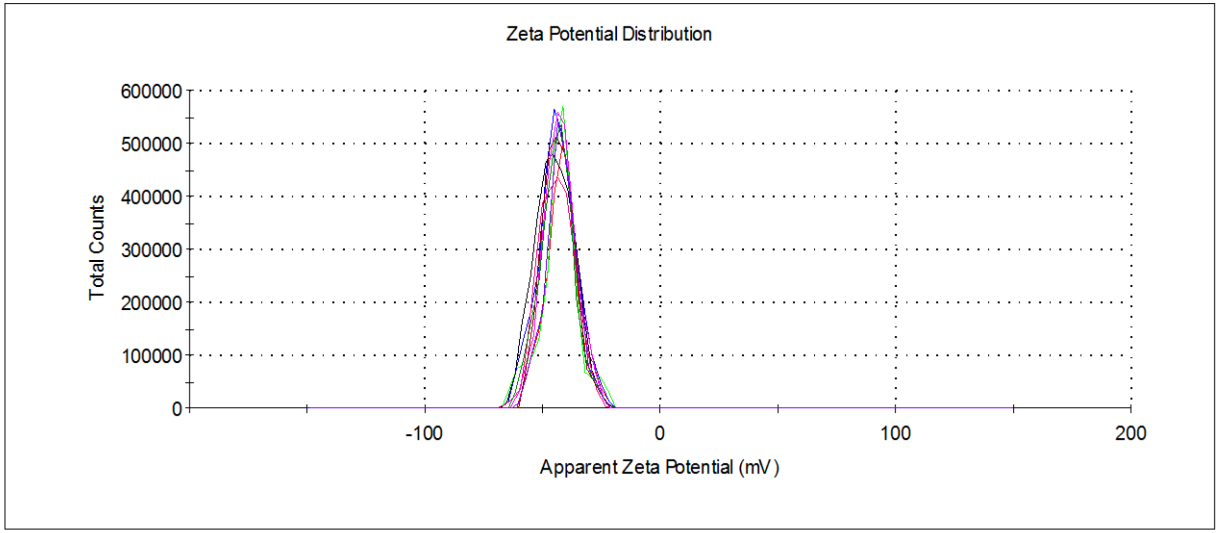

Fig. S39. Size distribution of the particles by intensity for SLN-5-DTAC (1×10^{-4} M) at 1:1000 ratio in water (the each colored line on the graph means one repeated measurement).

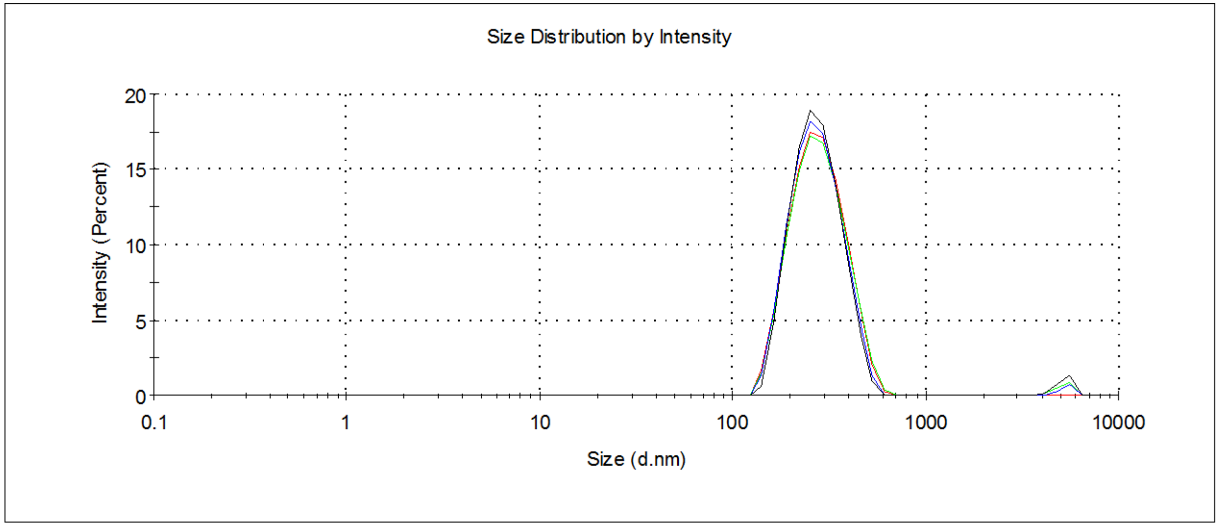

Fig. S40. Zeta potential distribution of the particles for SLN-5-DTAC (1×10^{-4} M) at 1:1000 ratio in water(the each colored line on the graph means one repeated measurement).

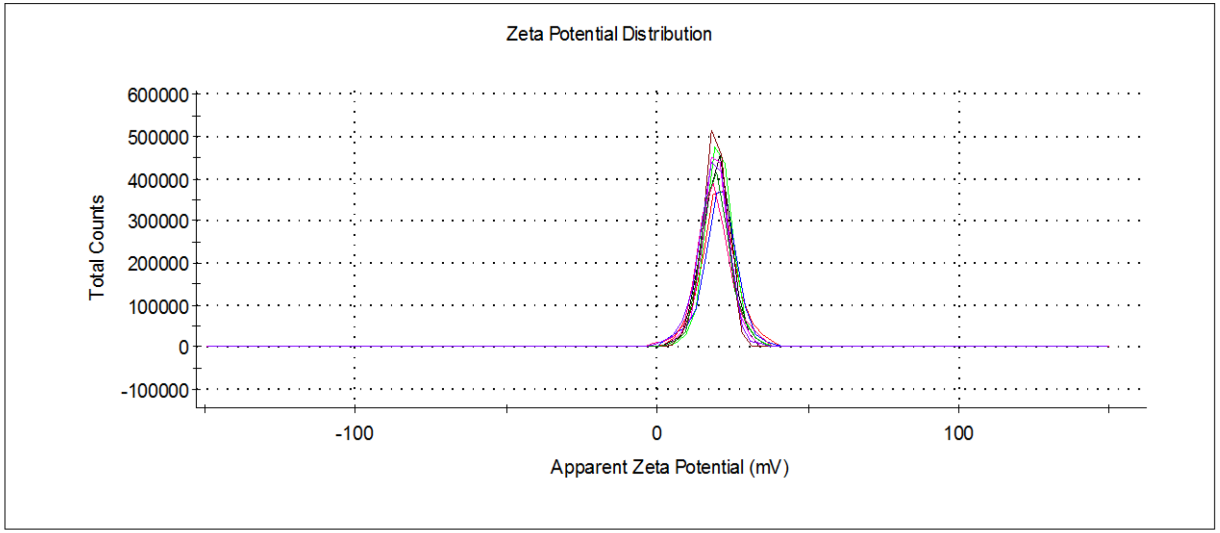

Fig. S41. Size distribution of the particles by intensity for SLN-6-DTAC (1×10^{-4} M) at 1:1 ratio in water (the each colored line on the graph means one repeated measurement).

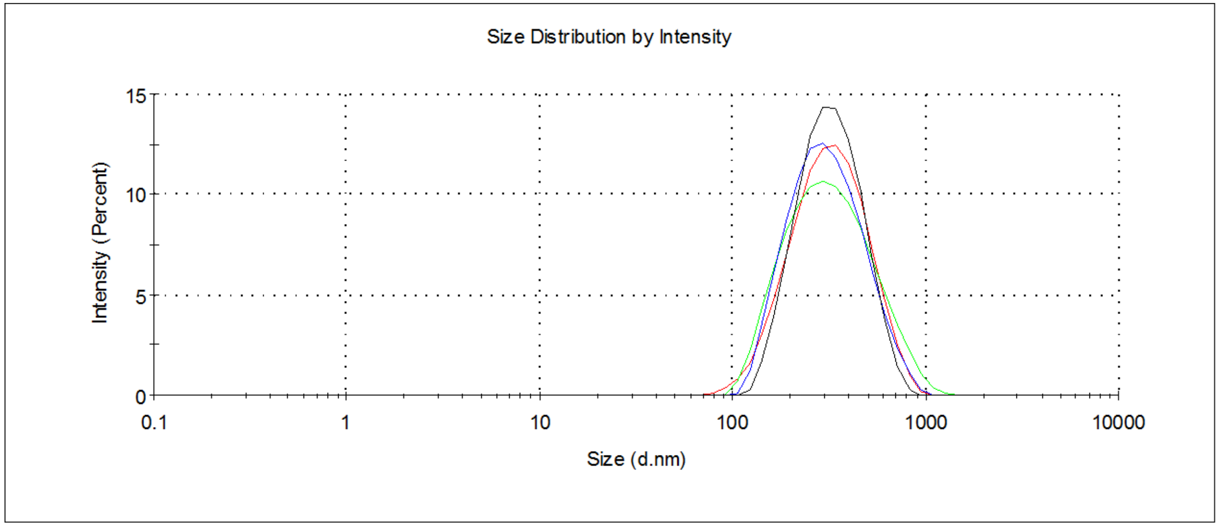

Fig. S42. Zeta potential distribution of the particles for SLN-6-DTAC (1×10^{-4} M) at 1:1 ratio in water (the each colored line on the graph means one repeated measurement).

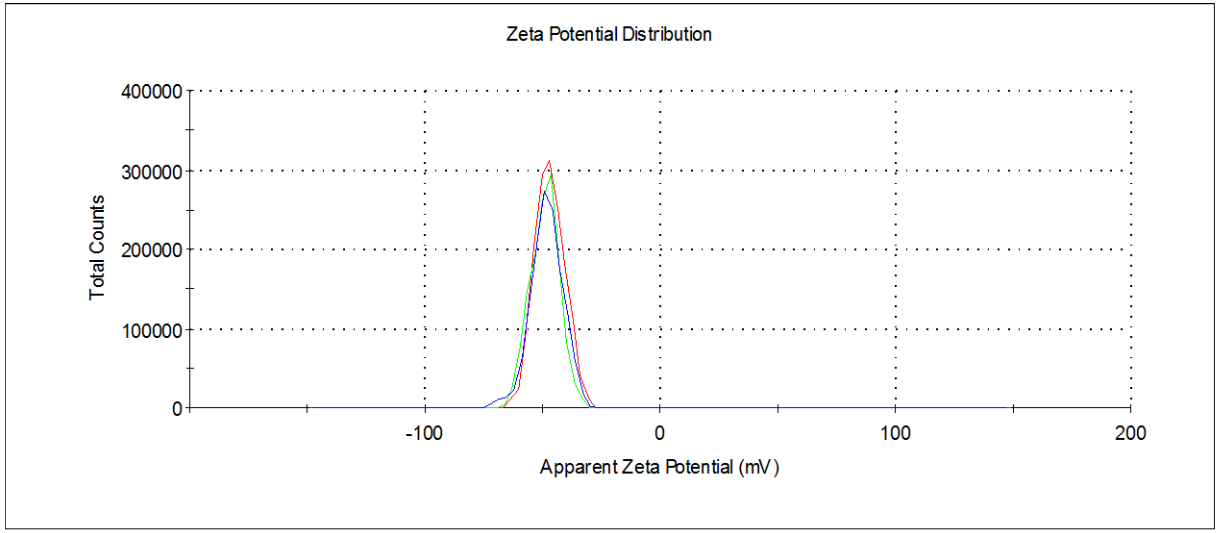

Fig. S43. Size distribution of the particles by intensity for SLN-6-DTAC (1×10^{-4} M) at 1:100 ratio in water.

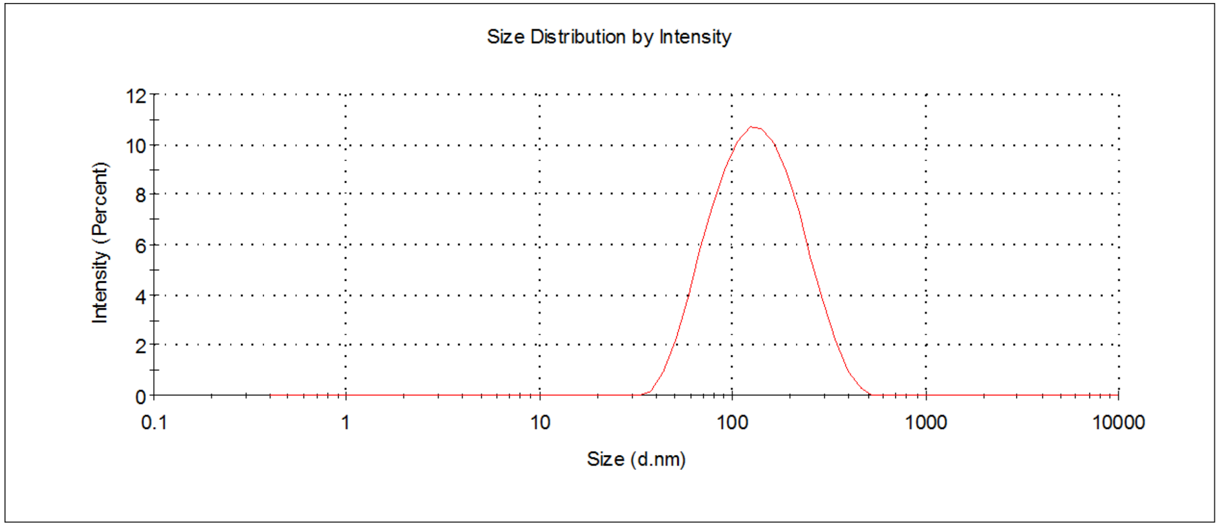

Fig. S44. Zeta potential distribution of the particles for SLN-6-DTAC (1×10^{-4} M) at 1:100 ratio in water.

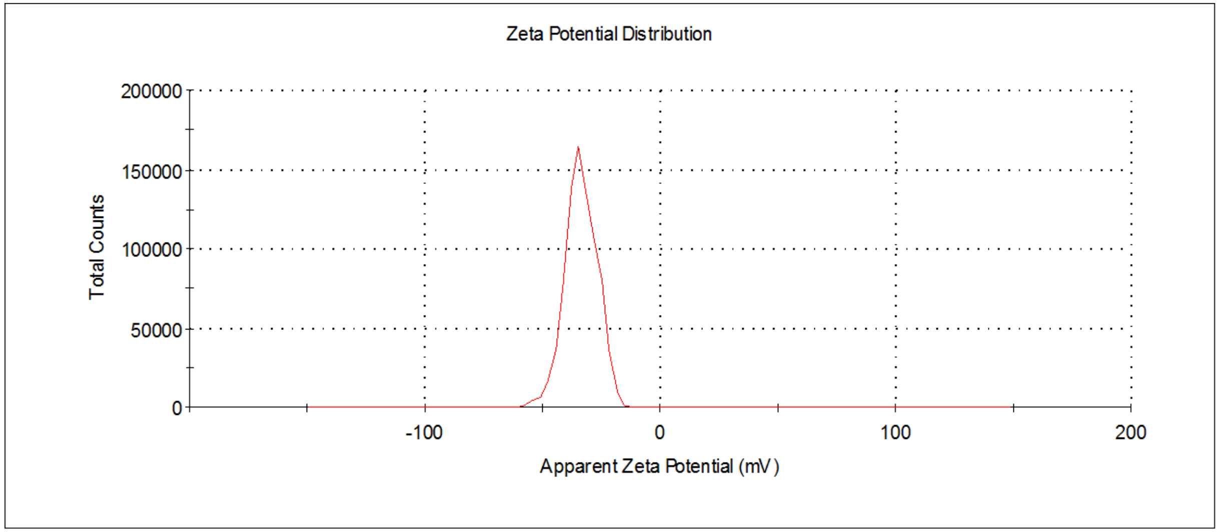

Fig. S45. ^{1}H NMR spectra (MeOD-d_4, 298 K, 400 MHz): a) dodecyltrimethylammonium chloride (0.005 mol/l); b) 5 (0.005 mol/l) + dodecyltrimethylammonium chloride (0.005 mol/l); c) 5 (0.005 mol/l).

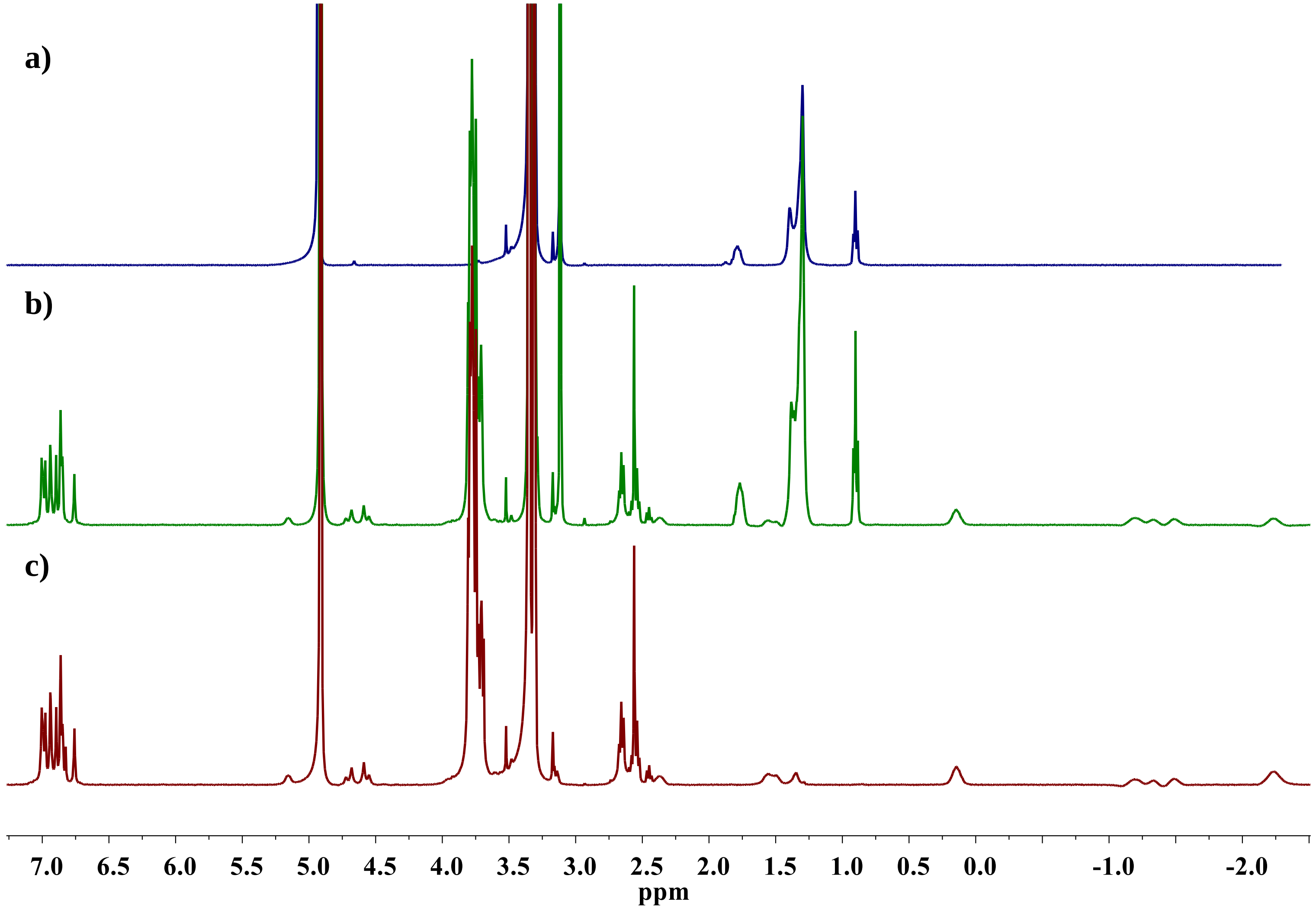

Fig. S46. ^{1}H **NMR spectra (MeOD-d_4, 298 K, 400 MHz): a) dodecyltrimethylammonium chloride (0.005 mol/l); b) 6 (0.005 mol/l) + dodecyltrimethylammonium chloride (0.005 mol/l); c) 6 (0.005 mol/l).**

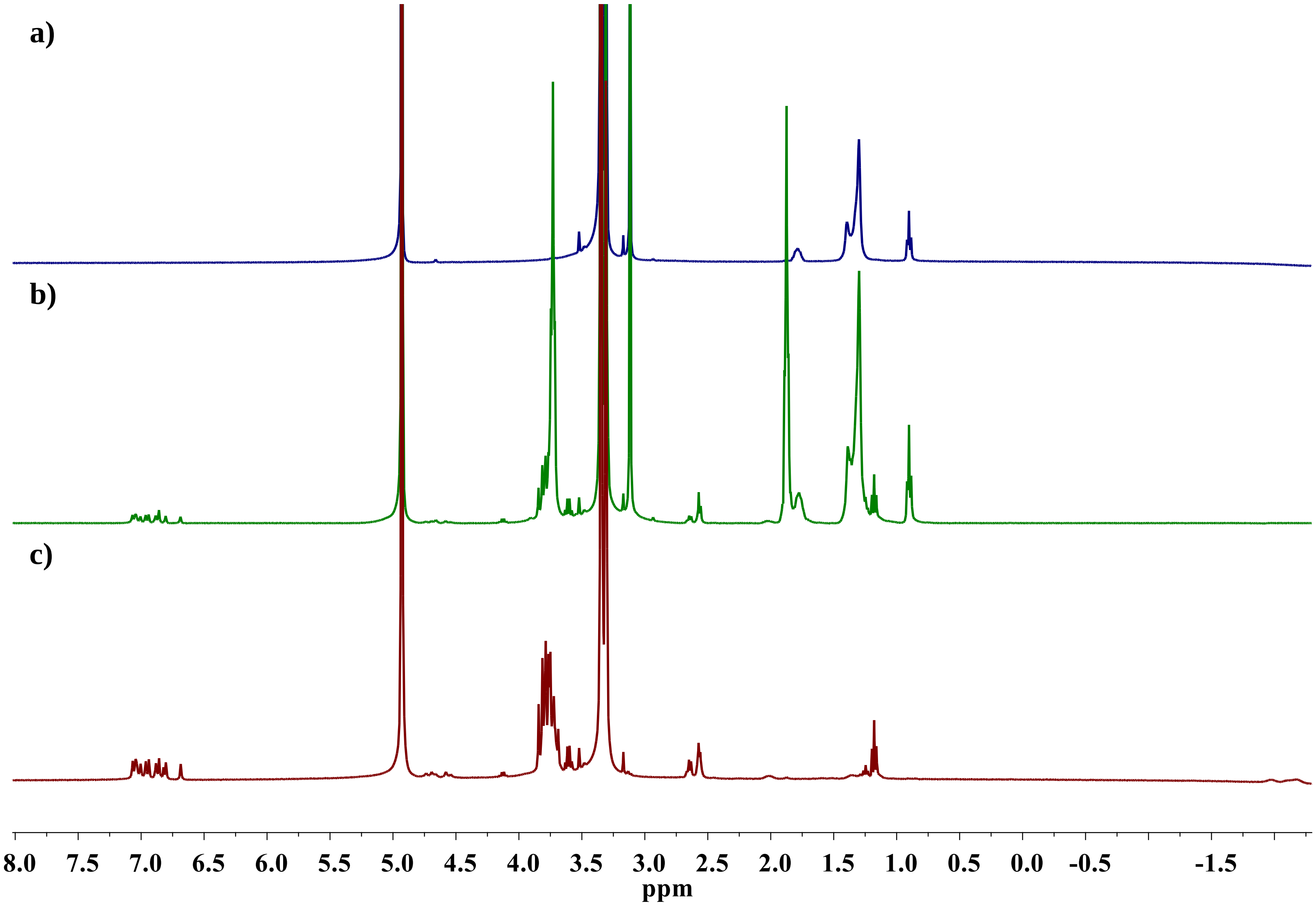

Supplement: Supplementary file 1 [file ijms-23-00779-s001.zip › ijms-1537760-supplementary.pdf]
